# Supplementary material for: The reaction of methyl peroxy and hydroxyl radicals as a major source of atmospheric methanol
Source: Nat Commun. 2016 Oct 17;7:13213. doi: 10.1038/ncomms13213 (PMC5071643; doi:10.1038/ncomms13213)
Supplement: Supplementary Information — Supplementary Figures 1-18, Supplementary Tables 1-7, Supplementary Notes 1-6 and Supplementary References [file ncomms13213-s1.pdf]

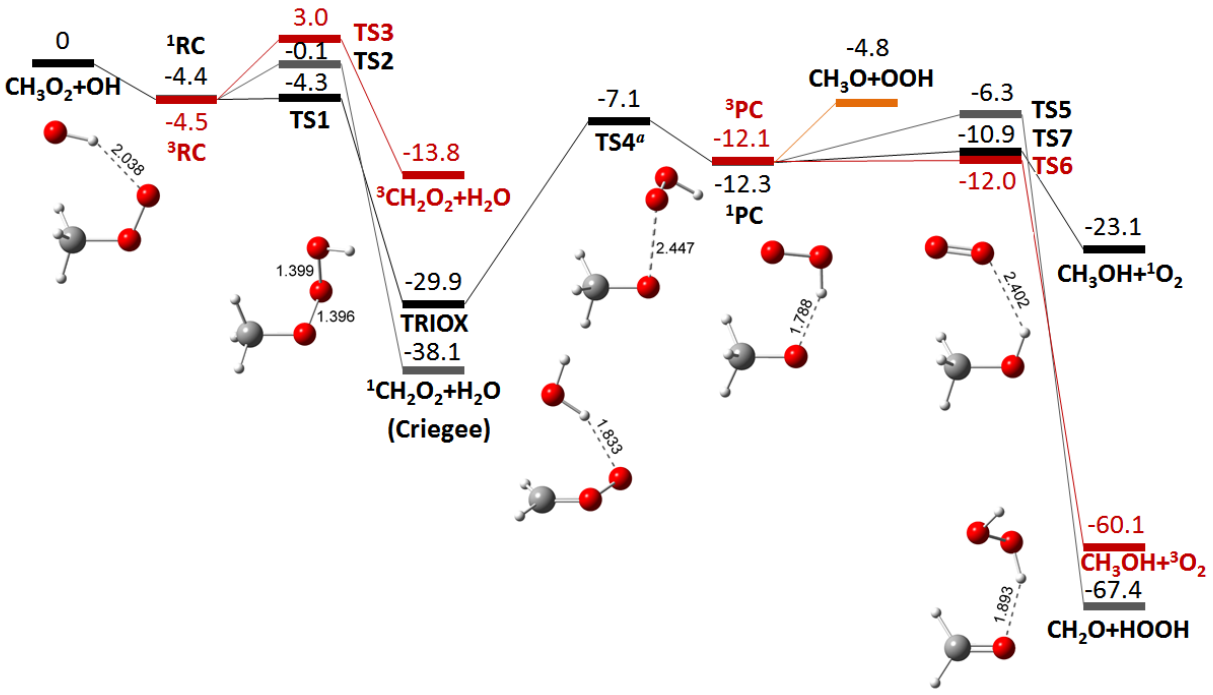

Supplementary Figure 1 | Potential energy surface for CH_3OO + OH biradical reactions. The geometries are optimized at M06-2X-D3/6-311++G(3df,3pd) level of theory and the energies (kcal mol^{-1}) with inclusion of ZPVE are relative to separated reactants CH_3O_2 and OH. The singlet reaction pathways are depicted in black and grey, while the triplet reaction pathways are depicted in red for clarity. [a]TS4 is optimized at M06-D3/6-311++G(3df,3pd) level of theory, see text.

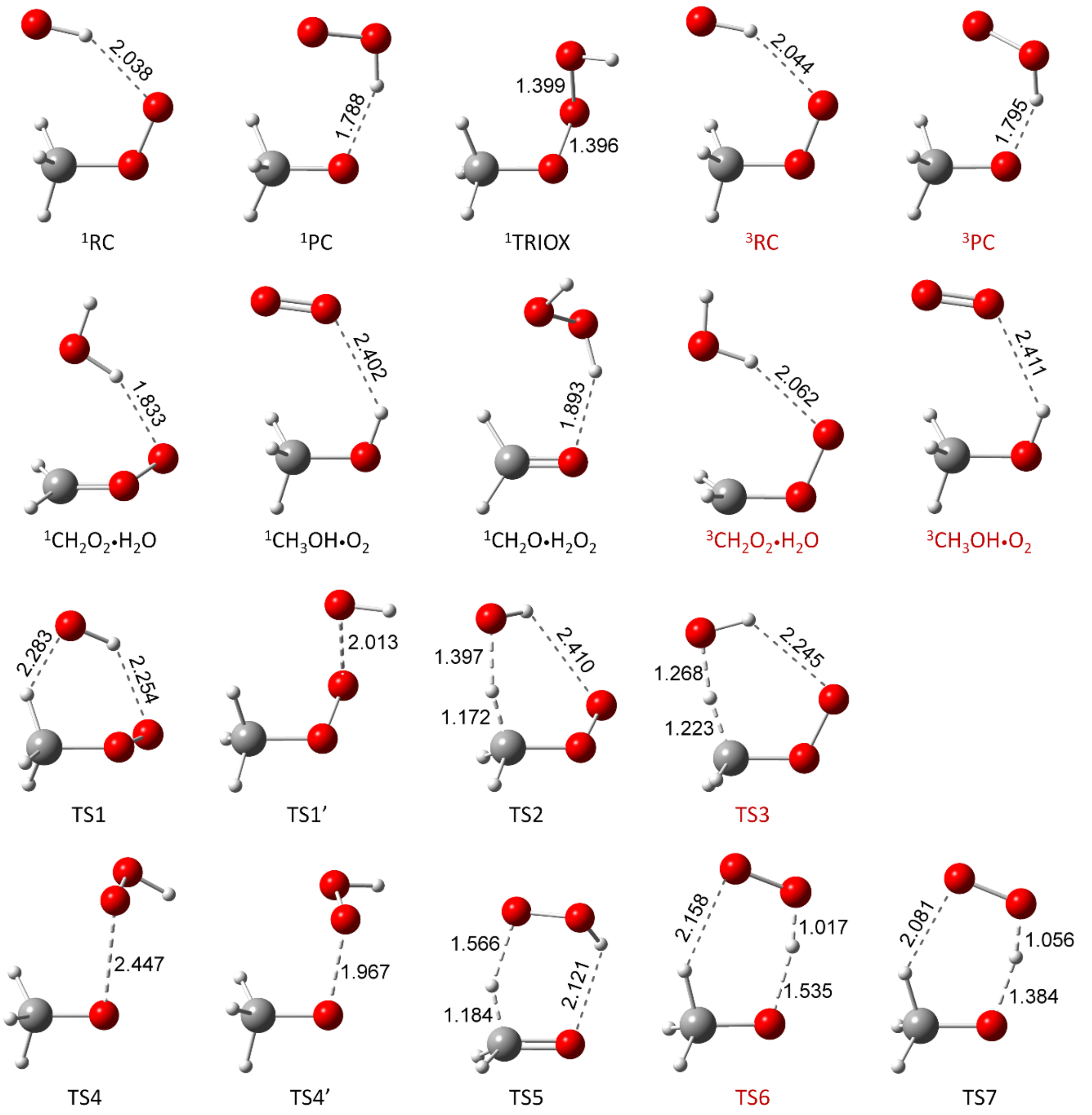

Supplementary Figure 2 | Various structures on the Potential energy surface. Geometries are optimized at M062x-D3/6-311++G(3df,3pd) level of theory. The singlet complexes are named in black and grey, while the triplet complexes are named in red for clarity. TS4 is optimized at M06-D3/6-311++G(3df,3pd) level of theory, see text. Bond distances are in angstrom.

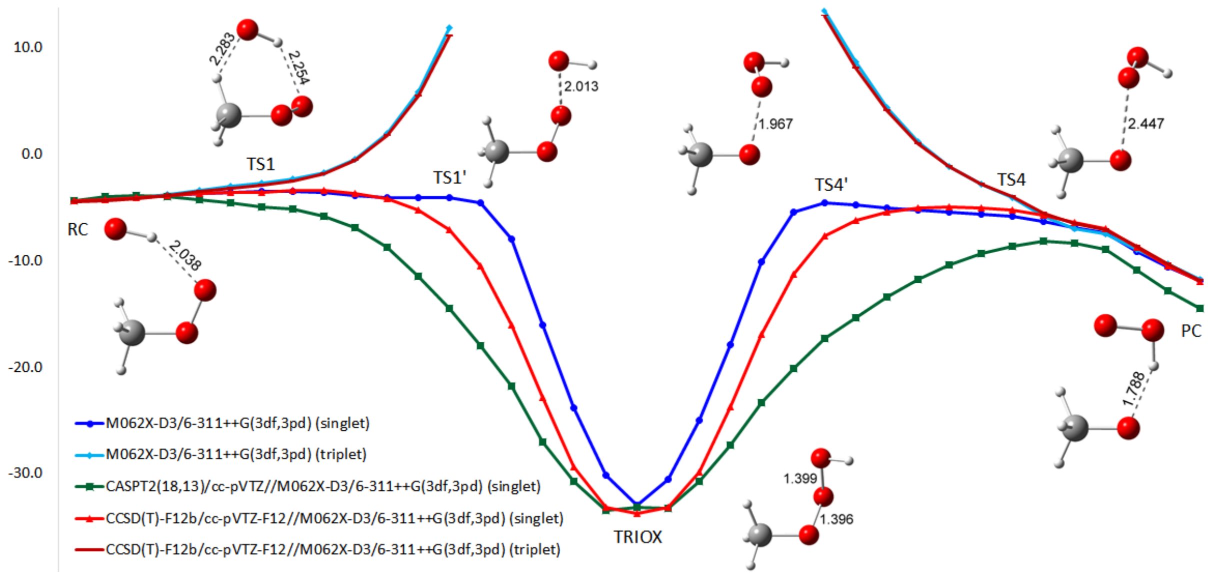

Supplementary Figure 3 | Rigid scan at various levels of theory for the pathway from the reactant complex via TRIOX to form the product complex. RC, **TS1**, **TS1'**, **TS4'**, and **PC** are optimized at M062x-D3/6-311++G(3df,3pd) level of theory, while **TS4** is optimized at M06-D3/6-311++G(3df,3pd) level of theory. CASPT2 and CCSD(T)-F12 calculations are carried out using DFT optimized structures and the structures obtained using linear interpolation of these stationary points. See Supplementary Note 1 for the detailed description. The energies (kcal mol^{-1}) without inclusion of ZPVE are relative to separated reactants CH_3O_2 and OH.

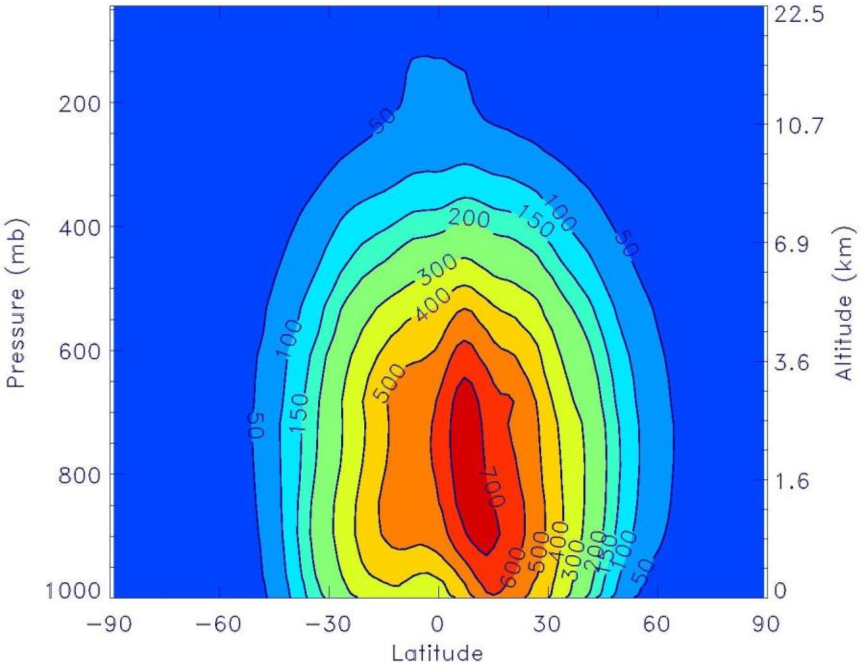

Supplementary Figure 4 | Annually and zonally averaged sink of CH_3O_2 through the CH_3O_2+OH reaction, in pptv/day. Calculated with the IMAGES model (see Methods section) assuming a rate constant of 2.8×10^{-10} molec.$^{-1}$ cm^3 s^{-1}.

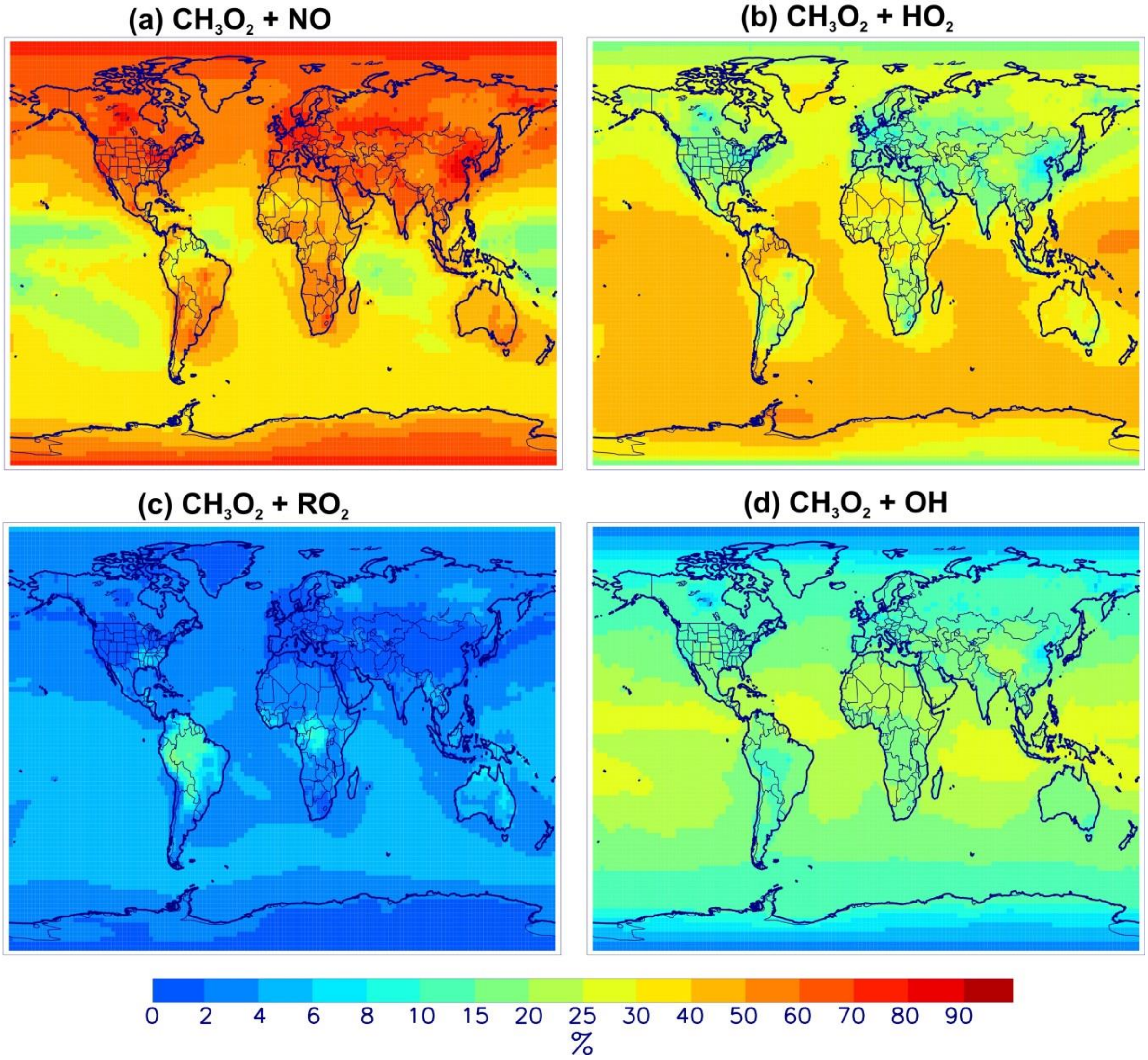

Supplementary Figure 5 | Relative contributions to the total sink of CH_3O_2. Model-calculated annually averaged contributions of the reactions of CH_3O_2 with (a) NO, (b) HO_2, (c) RO_2 and (d) OH to the sink of CH_3O_2 (vertical integral). The globally and annually averaged contributions of those four pathways are 43%, 33%, 4% and 20%, respectively. Maps created using IDL® version 8.2.3.

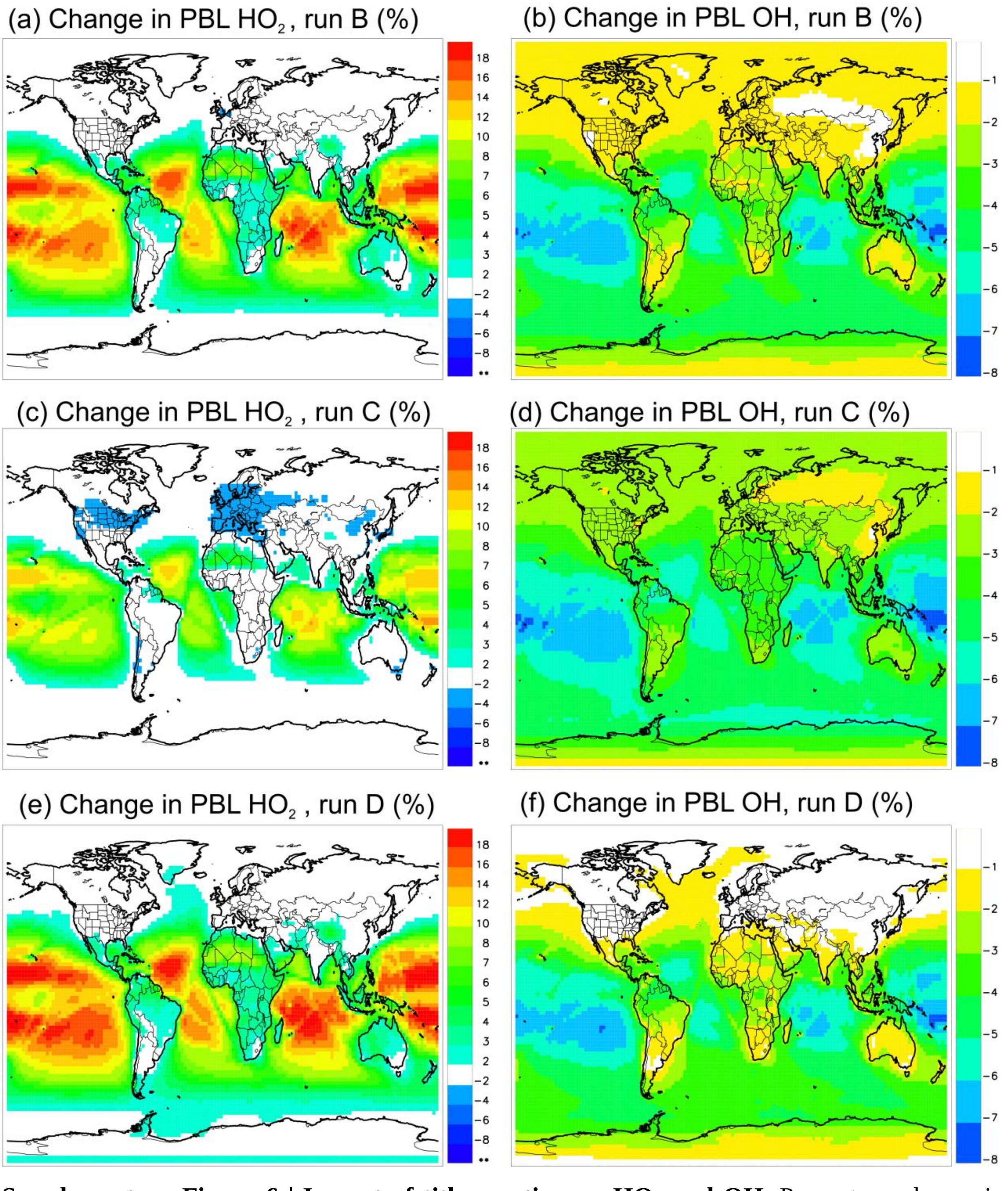

Supplementary Figure 6 | Impact of title reaction on HO_2 and OH. Percentage change in the annually averaged mixing ratio of HO_2 (left) and OH (right) in the planetary boundary layer (PBL) due to the CH_3O_2+OH reaction as implemented in run B (best yield estimates, top panels), in run C (high methanol case, middle panels) and in run D (low methanol case, bottom panels). Differences defined as e.g. ((B-A)/A)×100. The PBL is here the lowermost 1 km above surface. Maps created using IDL® version 8.2.3.

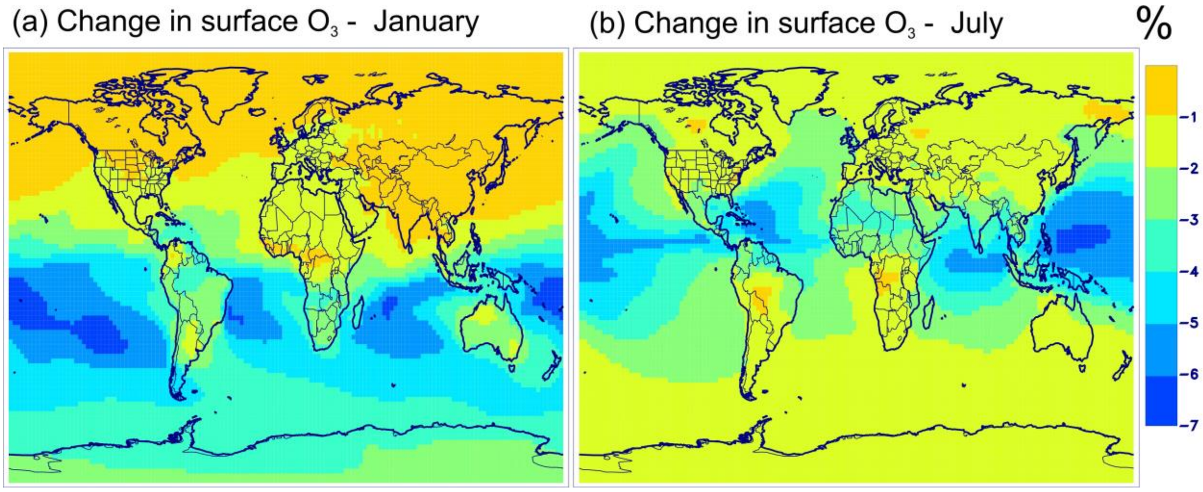

Supplementary Figure 7 | Impact of CH_3O_2+OH on near-surface ozone. Percentage change in the near-surface mixing ratio of ozone in January (left) and July (right) due to the CH_3O_2+OH reaction as implemented in run C (high methanol case). Differences defined as ((C-A)/A)×100. The changes are slightly larger in the simulations B and D, due to their higher HO_2 yields. Maps created using IDL® version 8.2.3.

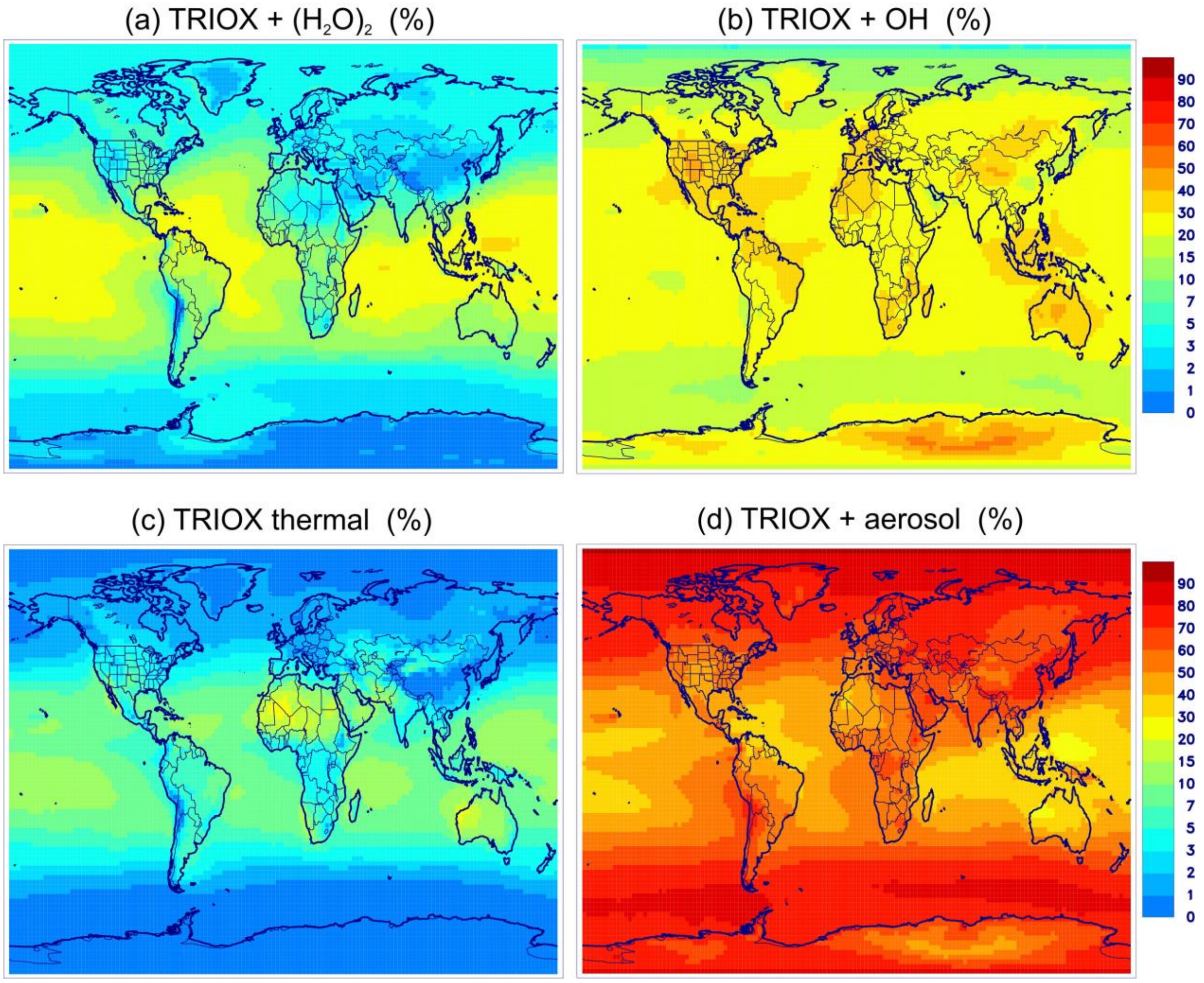

Supplementary Figure 8 | Relative contributions to the stabilized trioxide sink. Model-calculated yearly averaged contribution (%) of (a) the reaction with the water dimer, (b) reaction with OH, (c) thermal reaction and (d) reaction with aerosols to the vertically-integrated sink of the stabilized trioxide CH_3OOOH (TRIOX). The TRIOX reaction rates and products are described in the Supplementary Note 4. Maps created using IDL® version 8.2.3.

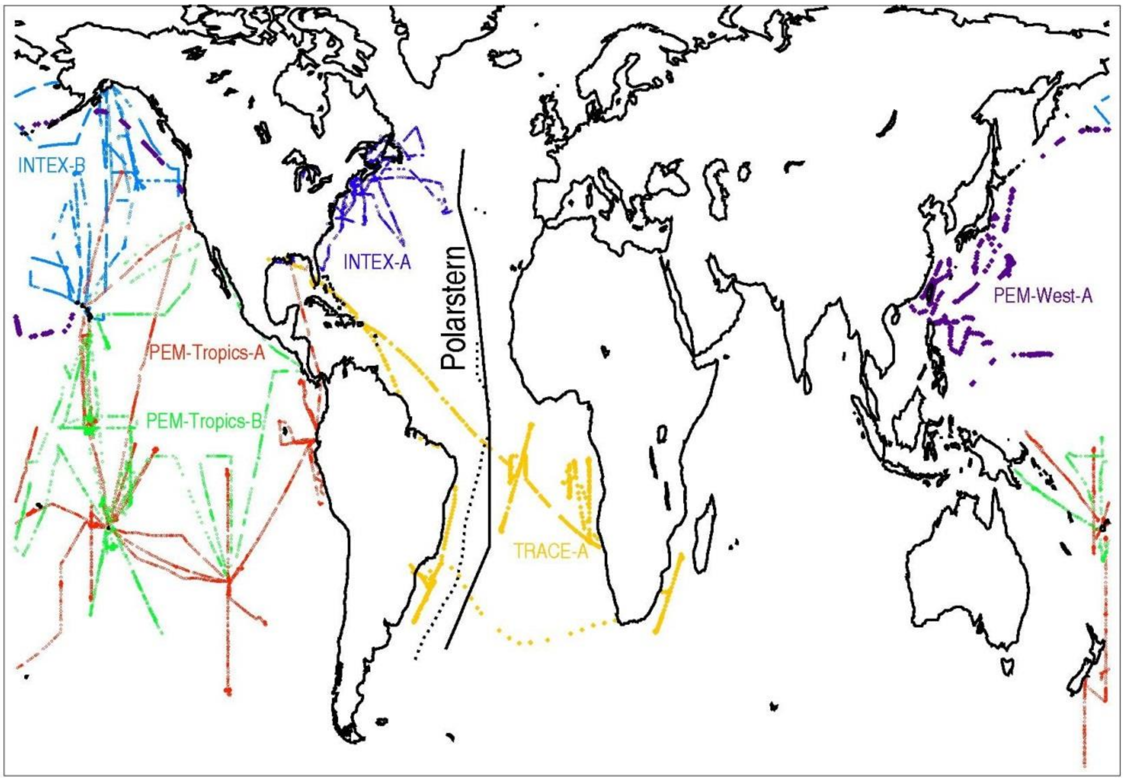

Supplementary Figure 9 | Atmospheric measurements used to evaluate the model results for peroxides and formaldehyde. Tracks of aircraft and ship measurements used for model evaluation of peroxides and formaldehyde. The aircraft campaigns include PEM-Tropics-A[1] conducted in August-October 1996, PEM-Tropics-B[2] in March-April 1999, PEM-West-A[1] in September-October 1991, INTEX-A[3] in July-August 2004, and INTEX-B[4] (DC8) in April-May 2006. Only measurements over ocean are shown, since only those were considered for model evaluation. Data obtained from www-air.larc.nasa.gov/missions/merges/. The Polarstern cruise tracks between 40°S and 49°N are also shown: the cruise of October/November 1994[5] (dotted black line) and the cruise of October/November 1996[6] (solid line). The measurement technique for peroxides during aircraft campaigns was high-performance liquid chromatography (HPLC), with an estimated accuracy of 30%. An enzyme fluorimetric technique was used in the Polarstern cruises as well as HPLC in the 1996 cruise. For formaldehyde, HPLC was used during PEM-Tropics-B (accuracy 15%), and Tunable Diode Laser absorption spectrometry was used by the NCAR team (accuracy 10%) during INTEX-A and INTEX-B. Map created using IDL® version 8.2.3.

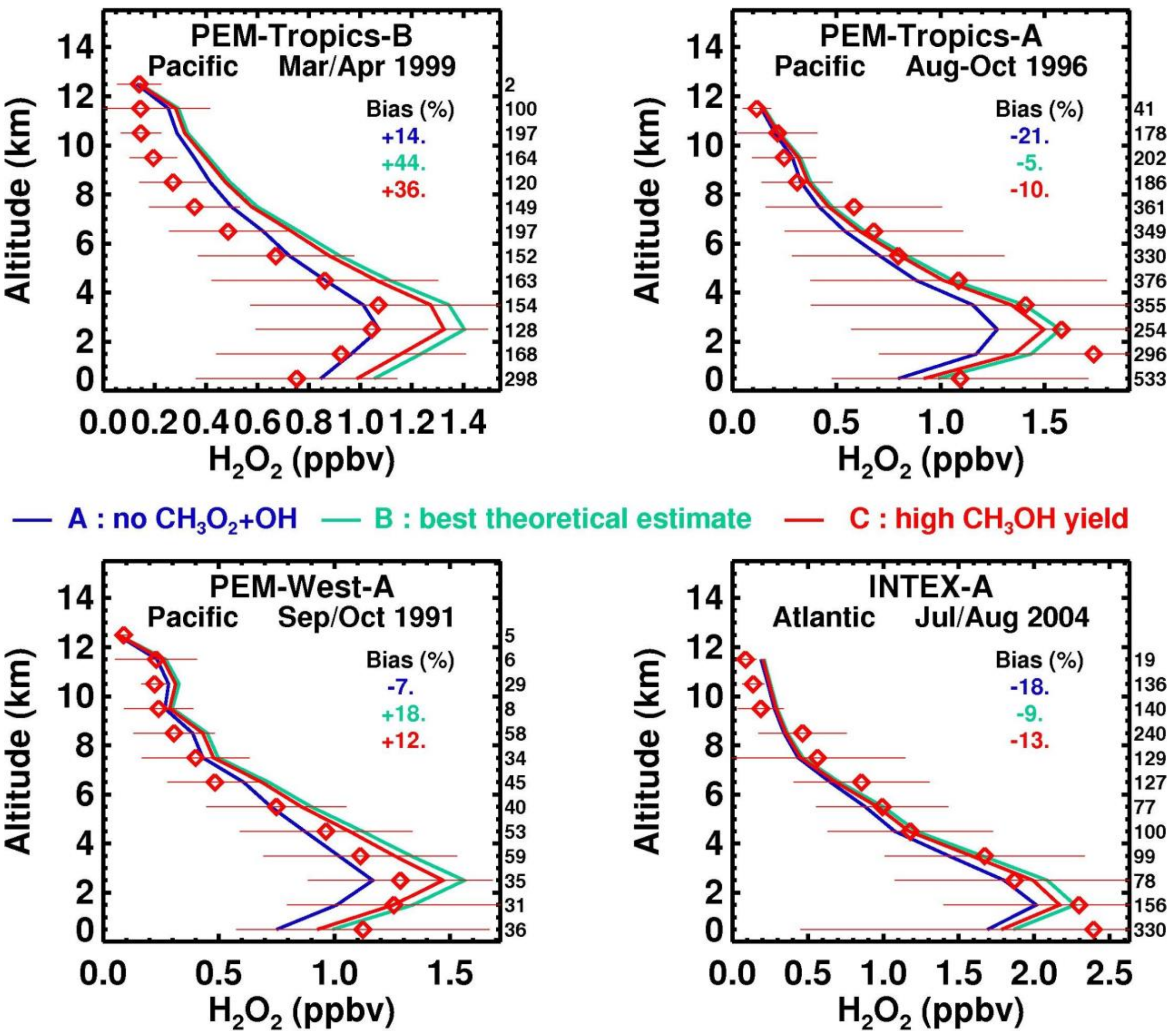

Supplementary Figure 10 | Measured and modelled H_2O_2 vertical profiles. Model results for simulations A, B, and C defined in Table 4 of main article. The flight tracks are shown on the previous Figure. Averaged volume mixing ratios of H_2O_2 in 1-km altitude bins during aircraft campaigns over the Pacific and Atlantic Oceans. The number of measurements at each altitude bin is indicated on the right of each plot. Error bars represent the standard deviations of the measurements. The average biases are also indicated inset.

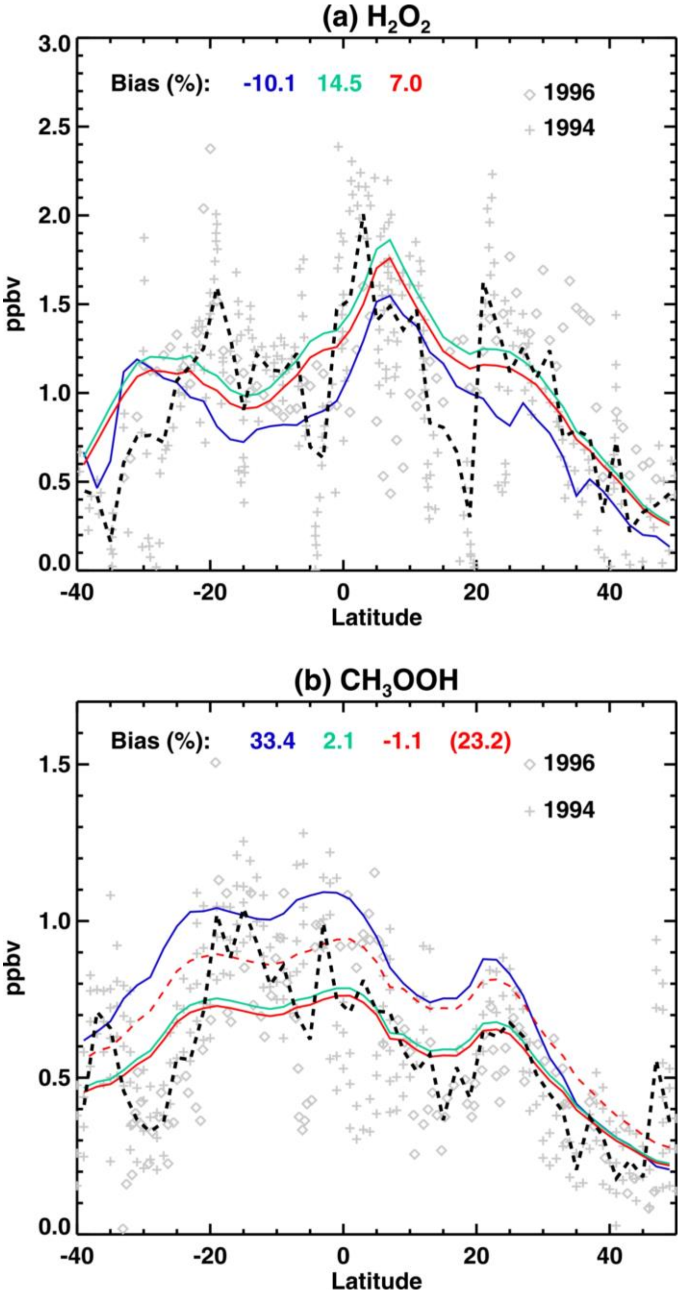

Supplementary Figure 11 | Peroxides during Polarstern cruises. Measured and modelled mixing ratios of (a) H_2O_2 and (b) CH_3OOH during the cruise of Oct./Nov. 1994[5] (crosses) and Oct./Nov. 1996[6] (diamonds). Itinerary used for sampling model results (Supplementary Fig. 9) is from Figure 1 in Weller et al.[6] (adopting the 1994 itinerary leads to similar results). The HPLC measurements from the 1996 cruise were excluded since those data showed very high sample-to-sample variability. Inclusion of the HPLC data would not alter the conclusions from these comparisons. Model results for simulations A (dark blue), B (green), and C (red) defined in Table 4 of main article. For CH_3OOH, the dashed red line corresponds to simulation C_VR, similar to C, adopting the lower measured rate constant for CH_3OOH+OH (see text). The measurements averaged on 2° latitude bins are shown as the dashed black line.

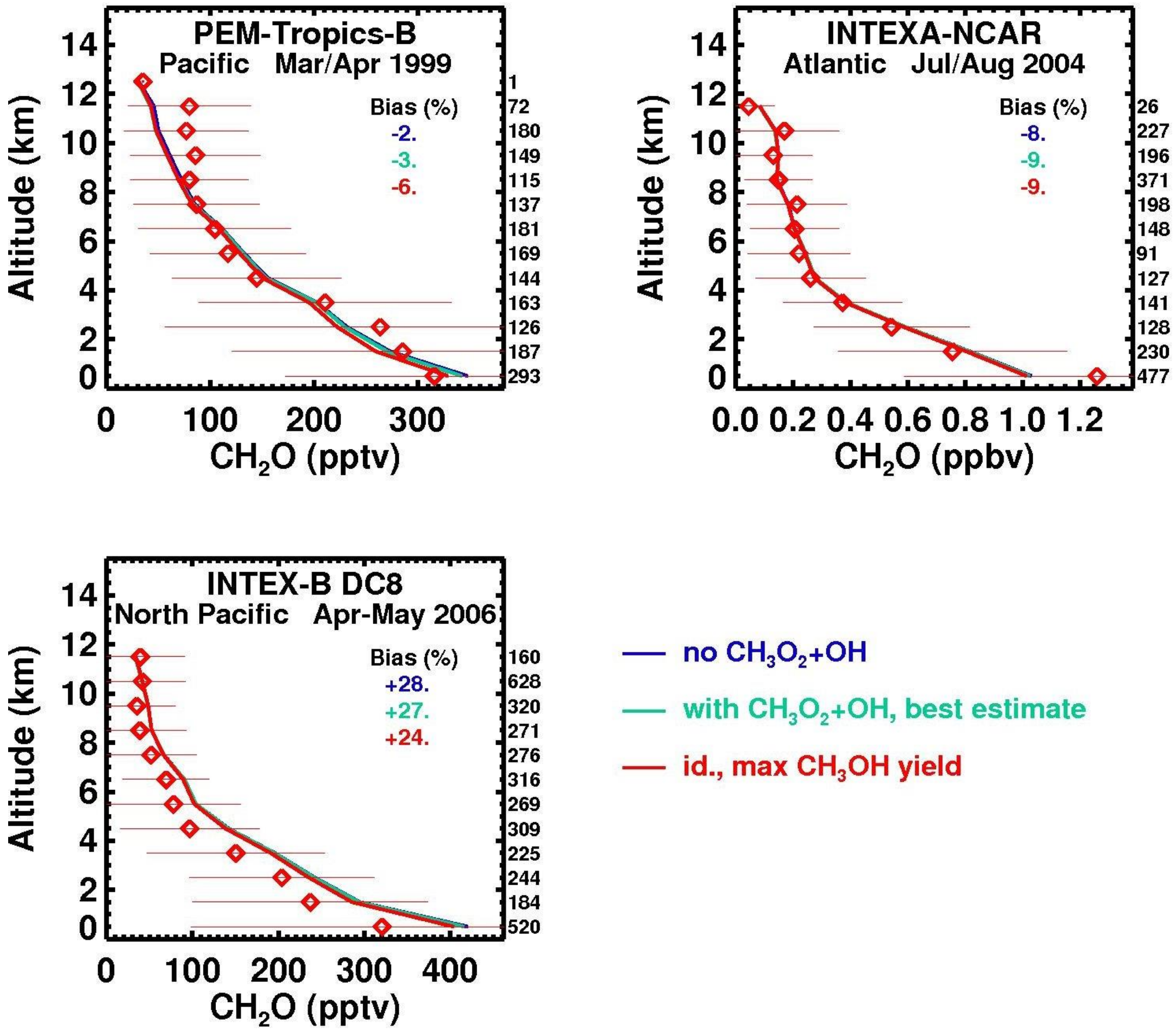

Supplementary Figure 12 | Observed and modelled CH_2O in aircraft campaigns. Model results for simulations A, B, and C defined in Table 4 of main article. The flight tracks are shown on Supplementary Figure 9. Vertical profiles of measured and modelled CH_2O mixing ratios averaged in 1-km altitude bins during several aircraft campaigns. Only measurements over oceans were considered. The number of measurements at each altitude bin is indicated on the right of each plot. Error bars represent the standard deviations of the measurements. The average biases are also indicated inset.

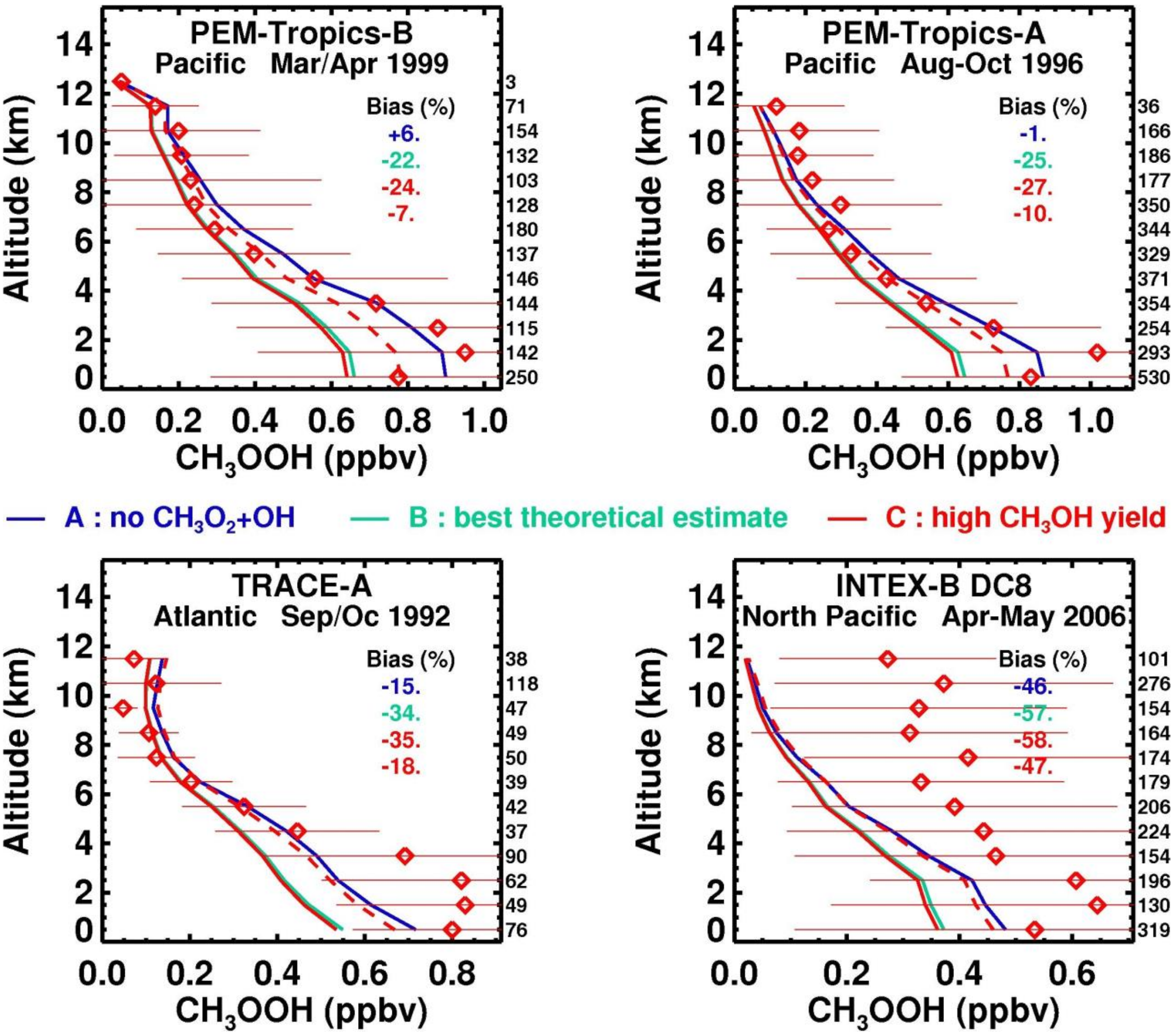

Supplementary Figure 13 | Observed and modelled CH_3OOH in aircraft campaigns. Continuous lines are model results for simulations A, B, and C (Table 4 of main article). The dashed red line is for a run C_VR similar to C, using the lower measurement of the rate constant for CH_3OOH+OH (see text). The flight tracks are shown on Supplementary Figure 9. Vertical profiles of measured and modelled CH_3OOH mixing ratios (ppbv) averaged in 1-km altitude bins during several aircraft campaigns over the Pacific. The number of measurements at each altitude bin is indicated on the right of each plot. Error bars represent the standard deviations of the measurements. The average biases are also indicated inset.

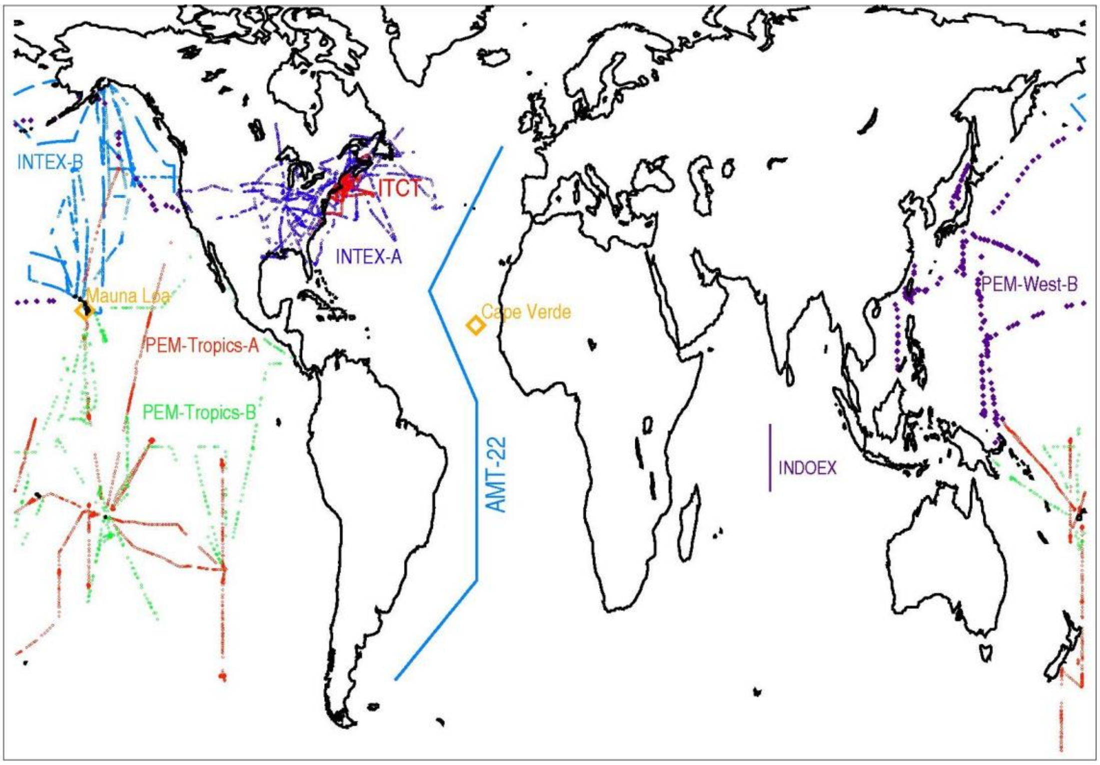

Supplementary Figure 14 | Atmospheric measurements used to evaluate the model results for methanol and formic acid. Locations of the aircraft and ship measurements used for model evaluation of CH_3OH and HCOOH. The aircraft campaigns include PEM-Tropics-A[7] in August-October 1996 (for HCOOH), PEM-Tropics-B[8] in March-April 1999, PEM-West-B[9] in February-March 1994, INTEX-A[3] in July-August 2004, ITCT/ICARTT[10] in July-August 2004, INTEX-B/MILAGRO (DC8)[4] in March 2006 and INTEX-B (DC8)[4] in April/May 2006. The data were obtained from www-air.larc.nasa.gov/missions/merges/. Methanol measurements also include the ship cruises AMT-22[11] in October-November 2012 and INDOEX-1999[12] in March 1999 (only Southern Hemisphere measurements were considered to limit continental influence), as well as measurements at Mauna Loa, Hawaii[13] in March/April 2001 and Cape Verde Atmospheric Observatory[14] between 2006 and 2011. Map created using IDL® version 8.2.3.

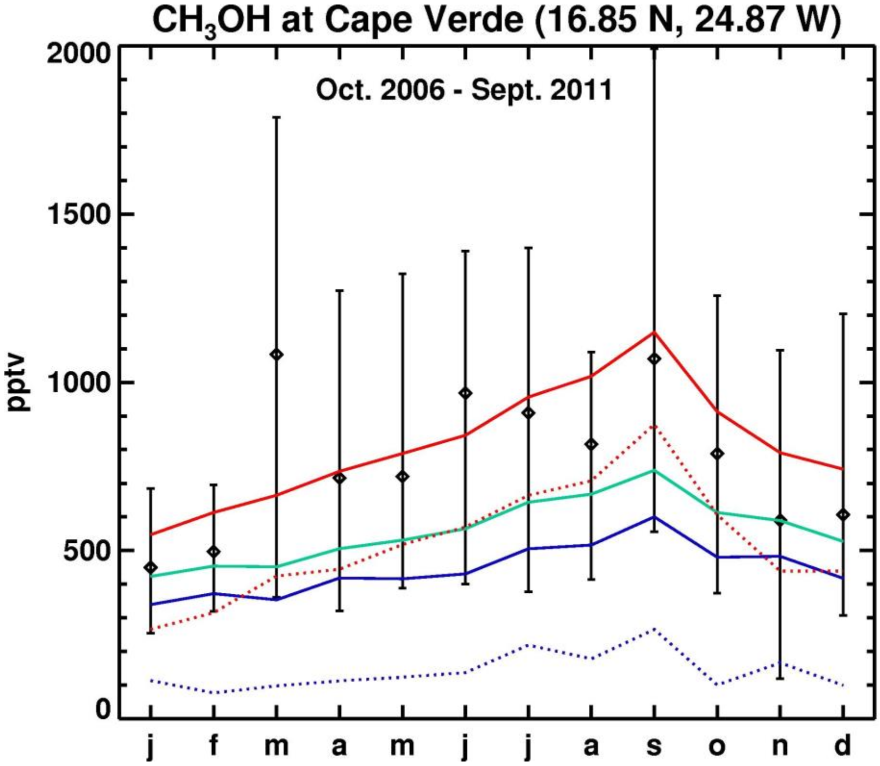

Supplementary Figure 15 | Observed and modelled methanol at Cape Verde. Monthly averaged mixing ratios measured at Cape Verde Atmospheric Observatory[14] (between October 2006 and September 2011) and simulated by the model in 2010. Monthly averaged observations and their 10th and 90th percentiles (represented as error bars) are from Figure 2 in Read et al.[14] Solid (dotted) lines denote simulations including (excluding) oceanic emissions of methanol. Deep blue: run A (no CH_3O_2+OH); green: run B (best theoretical estimates of the yields); red: run C (high overall methanol yield).

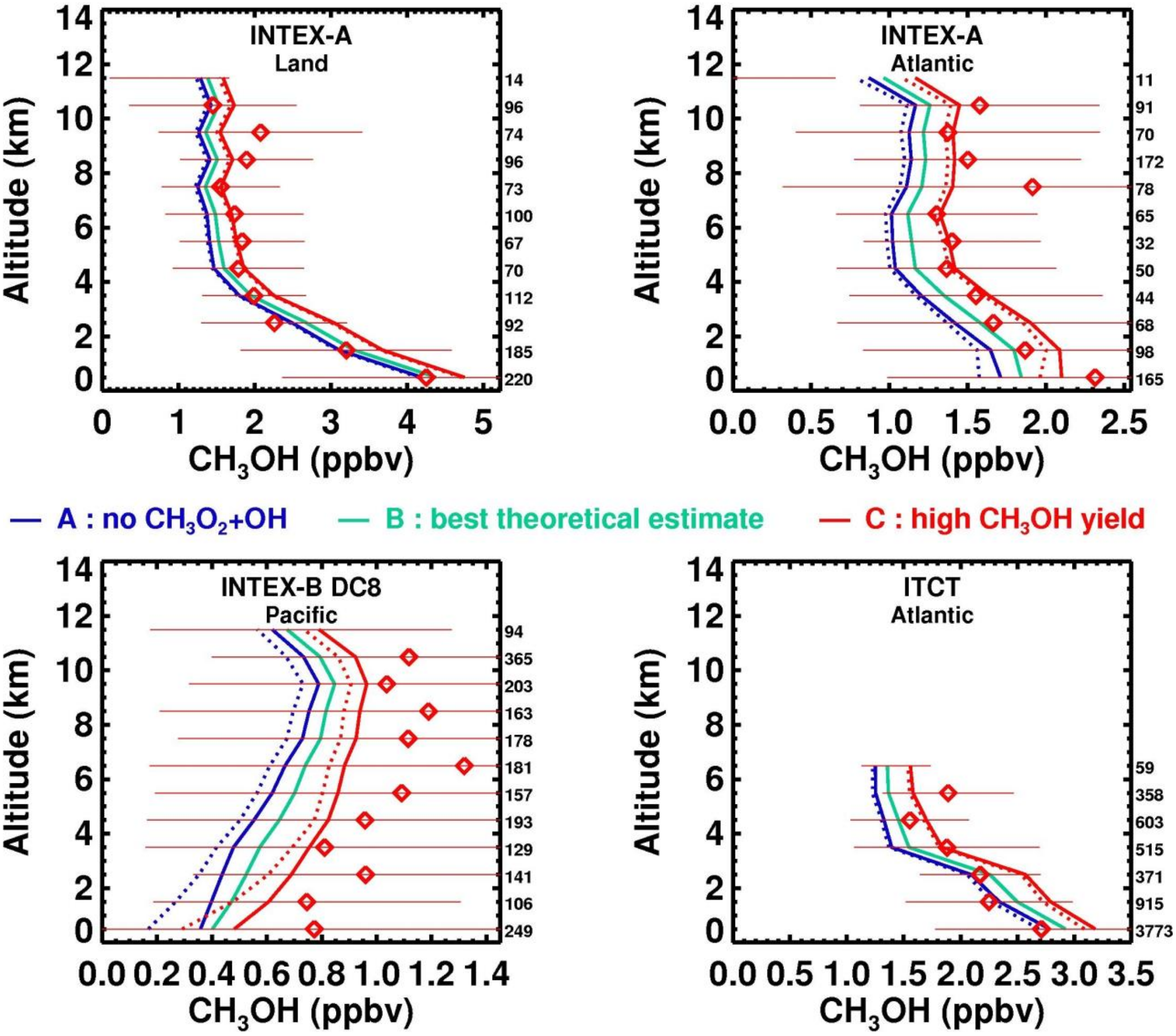

Supplementary Figure 16 | Observed and modelled CH_3OH in aircraft campaigns at mid-latitudes. Model results for simulations A, B, and C defined in Table 4 of main article. The flight tracks are shown on Supplementary Figure 14. Vertical profiles of measured and modelled CH_3OH mixing ratios (ppbv) averaged in 1-km altitude bins for several aircraft campaigns over the Pacific. The number of measurements at each altitude bin is indicated on the right of each plot. Error bars represent the standard deviations of the measurements.

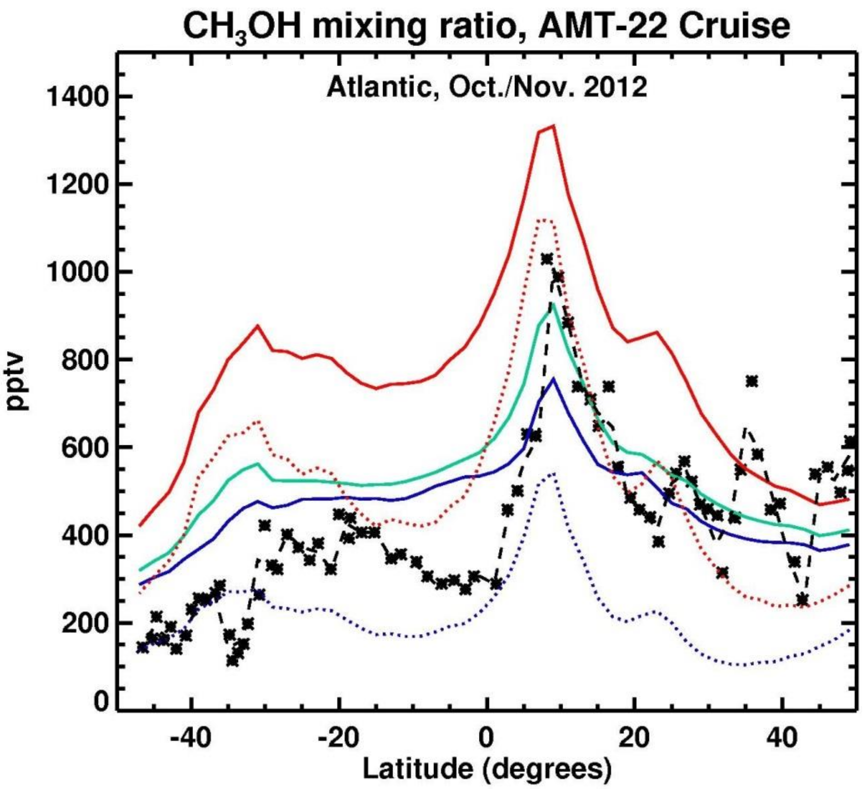

Supplementary Figure 17 | Measured and simulated methanol during cruise AMT-22[11]. Ship itinerary (see previous Figure) obtained from Figure 1 in Yang et al.[11] Solid (dotted) lines denote simulations including (excluding) oceanic emissions of methanol. Deep blue: run A (no CH_3O_2+OH); green: run B (best theoretical estimates of the yields); red: run C (high overall methanol yield). The measurements averaged on 2° latitude bins are shown as the dashed line.

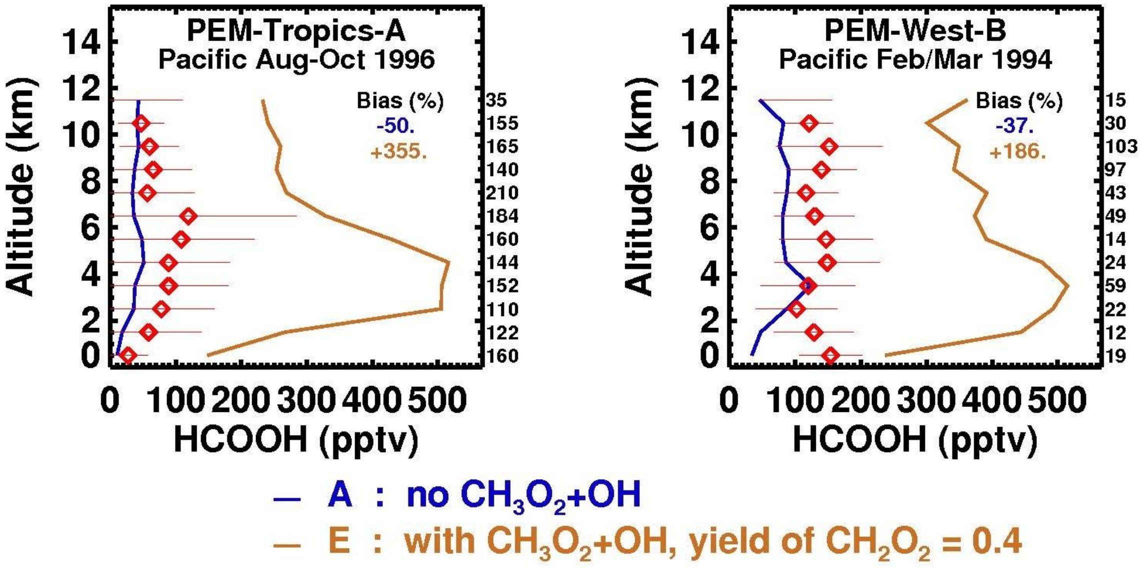

Supplementary Figure 18 | Evaluation of HCOOH using aircraft campaigns. Model results for simulations A and E (Table 4 of main article) illustrating the impact which a significant yield (0.4) of Criegee CH_2OO from CH_3O_2+OH would have on HCOOH over remote oceans. See Supplementary Figure 14 for information on the aircraft campaigns. The measured and modelled HCOOH (pptv) are averaged in 1-km altitude bins. Formic acid was measured by ion chromatography[15] with estimated precision and accuracy of 15% and a detection limit of 10-31 pptv[16]. The model includes HCOOH formation from the OH-reaction of vinyl alcohol, the latter being formed from the photo-tautomerization of acetaldehyde as implemented in Peeters et al.[17] The number of measurements at each altitude bin is indicated on the right of each plot. Error bars represent the standard deviations of the measurements. The average biases are also indicated inset.

Supplementary Table 1 | List of frequencies, zero-point energies, rotational constants, and relative CCSD(T)-F12 energies for various structures for the title reaction.

List of Frequencies (cm^{-1}) for various structures											
^{1}RC	^{3}RC	^{1}TRIOX	^{1}PC	^{3}PC	TS1	TS2	TS3	TS4	TS5	TS6	TS7
63	91	164	37	53	-123	-1110	-1608	-209	-1035	-398	-1052
125	129	217	121	120	72	122	37	72	146	99	97
174	173	386	138	139	117	188	178	115	208	172	170
199	200	460	166	155	137	286	273	130	241	257	239
404	364	605	242	245	192	337	362	162	351	267	332
415	403	920	538	576	280	512	480	251	400	721	589
527	528	993	812	853	521	780	716	968	679	884	968
974	973	1084	1036	1029	979	886	820	1016	1042	1024	1004
1142	1143	1188	1170	1149	1143	1038	990	1137	1222	1162	1164
1206	1206	1231	1295	1293	1205	1140	1100	1228	1236	1277	1186
1329	1328	1434	1357	1364	1326	1264	1187	1349	1271	1346	1344
1463	1463	1460	1407	1395	1463	1331	1243	1358	1324	1365	1368
1487	1487	1483	1506	1513	1484	1377	1311	1466	1496	1516	1515
1495	1495	1517	1553	1548	1491	1469	1462	1491	1524	1599	1563
3080	3080	3066	2955	2952	3079	1634	1475	2901	1754	2471	1728
3166	3034	3034	3031	3033	3033	3033	3031	3030	3030	3029	3030
3198	3105	3100	3113	3096	3093	3087	3085	3084	3082	3081	3081
3701	3594	3641	3449	3661	3672	3686	3691	3692	3693	3693	3693
CCSD(T)-F12 relative energies with inclusion of ZPE (kcal mol^{-1})											
-2.7	-2.8	-29.0	-10.7	-10.5	-2.6	1.0	4.6	-4.9	-4.8	-9.5	-8.1
T1 diagnostic for CCSD(T)-F12 calculations											
0.025	0.024	0.015	0.028	0.025	0.026	0.035	0.028	0.027	0.038	0.044	0.081
Rotational constants (GHZ)											
11.35	11.34	19.70	16.17	16.15	11.02	11.60	11.54	17.54	15.74	16.44	16.66
4.398	4.438	5.833	3.704	3.698	4.607	4.942	5.168	3.544	4.641	4.059	4.314
3.235	3.256	5.186	3.096	3.136	3.440	3.725	3.674	3.513	3.728	3.342	3.507

Supplementary Table 2 | List of frequencies, zero-point energies, rotational constants, and relative CCSD(T)-F12 energies for various structures on the dissociative path of singlet product complex (^{1}PC).

List of Frequencies (cm^{-1}) for ^{1}PC → CH_3O+OOH										
^{1}PC	O-H: 2.0	O-H: 2.2	O-H: 2.4	O-H: 2.6	O-H: 2.8	O-H: 3.0	O-H: 3.2	O-H: 3.4	O-H: 3.6	O-H: 3.8
37	45	42	-72	-100	-197	-163	-141	-105	-101	-81
121	128	77	37	31	37	37	47	48	48	45
138	133	130	120	110	94	73	63	55	50	51
166	151	131	129	123	104	100	94	92	87	92
242	173	146	140	132	137	133	132	122	108	101
538	448	370	301	252	159	145	137	130	130	130
812	791	763	733	706	769	736	725	728	749	768
1036	1036	1036	1036	1034	1013	1014	1013	1008	1001	992
1170	1164	1163	1162	1160	1146	1148	1148	1147	1145	1142
1295	1290	1287	1285	1283	1278	1276	1275	1275	1274	1273
1357	1361	1363	1365	1366	1372	1371	1369	1369	1369	1370
1407	1405	1406	1406	1405	1397	1398	1398	1396	1395	1392
1506	1506	1485	1472	1467	1475	1475	1474	1472	1470	1470
1553	1514	1510	1510	1511	1519	1518	1518	1518	1519	1520
2955	2958	2960	2962	2962	2958	2952	2948	2945	2942	2941
3031	3034	3034	3033	3033	3033	3031	3030	3030	3029	3030
3113	3105	3100	3096	3093	3087	3085	3084	3082	3081	3081
3449	3594	3641	3661	3672	3686	3691	3692	3693	3693	3693
CCSD(T)-F12 relative energies with inclusion of ZPE (kcal mol^{-1})										
-10.7	-10.2	-9.5	-8.7	-7.9	-6.9	-6.4	-6.0	-5.8	-5.6	-5.4
T1 diagnostic for CCSD(T)-F12 calculations										
0.028	0.027	0.027	0.027	0.027	0.026	0.026	0.026	0.026	0.026	0.026
Rotational constants (GHZ)										
16.174	16.103	16.154	16.266	16.406	15.582	15.359	15.344	15.420	15.592	15.863
3.704	3.495	3.308	3.129	2.958	3.094	3.078	2.995	2.883	2.760	2.633
3.096	2.950	2.815	2.686	2.563	2.771	2.813	2.765	2.678	2.573	2.463
Variational <k_{diss}> at 298 K and 1013 hPa = 2.43 × 10^{12} s-1 for r(O-H) = 3.2 A										

Supplementary Table 3 | List of frequencies, zero-point energies, rotational constants, and relative CCSD(T)-F12 energies for various structures on the dissociative path of triplet product complex (^{3}PC).

List of Frequencies (cm^{-1}) for ^{3}PC → CH_3O+OOH									
^{3}PC	O-H: 2.0	O-H: 2.2	O-H: 2.4	O-H: 2.6	O-H: 2.8	O-H: 3.0	O-H: 3.2	O-H: 3.4	O-H: 3.6
53	51	44	-73	-133	-186	-173	-183	-118	-103
120	119	81	35	22	46	46	34	20	31
139	138	112	112	106	95	79	44	57	52
155	145	132	122	112	106	101	68	81	74
245	177	144	136	128	139	142	123	127	117
576	466	372	303	215	163	144	141	156	150
853	843	820	791	801	789	755	306	608	686
1029	1027	1024	1022	1013	1009	1007	1053	1036	1016
1149	1149	1149	1150	1146	1145	1145	1178	1159	1148
1293	1290	1287	1284	1282	1279	1277	1275	1274	1273
1364	1366	1367	1368	1371	1373	1372	1363	1367	1369
1395	1396	1397	1398	1395	1395	1396	1420	1409	1401
1513	1509	1486	1472	1471	1475	1473	1473	1471	1469
1548	1515	1513	1513	1516	1519	1517	1507	1514	1517
2952	2956	2959	2960	2960	2959	2959	2964	2960	2957
3033	3039	3038	3037	3035	3034	3034	3023	3029	3030
3096	3103	3098	3094	3090	3086	3084	3086	3083	3081
3661	3594	3641	3662	3674	3687	3691	3693	3693	3693
CCSD(T)-F12 relative energies with inclusion of ZPE (kcal mol^{-1})									
-10.5	-10.1	-9.4	-8.6	-7.8	-6.9	-6.4	-6.5	-5.8	-5.6
T1 diagnostic for CCSD(T)-F12 calculations									
0.025	0.024	0.024	0.024	0.024	0.024	0.024	0.024	0.024	0.024
Rotational constants (GHZ)									
16.151	16.066	16.102	16.192	16.017	15.618	15.551	15.373	15.395	15.451
3.698	3.500	3.315	3.140	3.048	3.093	3.046	3.021	2.936	2.836
3.136	2.985	2.844	2.714	2.677	2.762	2.744	2.677	2.608	2.525
Variational $<^3k_{diss}>$ at 298 K and 1013 hPa = 3.37×10^{12} s^{-1} for r(O-H) = 3.0 A									

Supplementary Table 4 | Measured H_2O_2 over oceanic areas and mean biases of model simulations. Averaged observed mixing ratios (pptv) and ratios of averaged model values to averaged observed values for simulations (A, B, C) defined in Table 4. Only measurements over oceans were considered. See Supplementary Figure 9 for more information on the measurements. ***n*** is the number of measurements per campaign. The mean bias factor is the geometrically averaged ratio of modelled to observed averages, and the discrepancy factor is the geometrically averaged ratio of the higher to the lower among the modelled and observed averages, as also defined as in Table 5 of main article. Note that a perfect model would have the bias factor equal to 1 and the discrepancy factors equal to 1 + *s*, with *s* the average fractional error of the measurements.

**campaign**	**area**	***n***	**Obs. [ppt]**	**A**	**B**	**C**
PEM-Tropics-A	Pacific	3460	944	0.79	0.95	0.90
PEM-Tropics-B	Pacific	1990	591	1.14	1.44	1.36
TRACE-A	Atlantic	790	1206	0.76	0.85	0.82
PEM-West-A	W. Pacific	440	762	0.93	1.18	1.12
INTEX-A	N.E. Atlantic	1630	1222	0.81	0.91	0.87
INTEX-B DC8	N.E. Pacific	2260	751	0.76	0.84	0.80
Polarstern 1994	Atlantic	~300	915	0.94	1.19	1.12
Polarstern 1996	Atlantic	~200	1096	0.82	1.04	0.97
Mean bias factor				0.86	1.03	0.98
Mean discrepancy factor				1.20	1.17	1.17

Supplementary Table 5 | Measured CH_3OOH over oceanic areas and mean biases of model simulations. Averaged observed mixing ratios (pptv) and ratios of averaged model values to averaged observed values for simulations A, B, C and C_VR (Table 4). Only measurements over oceans were considered. See Supplementary Figure 9 for more information on the measurements. ***n*** is the number of measurements per campaign. The mean bias factor is the geometrically averaged ratio of modelled to observed averages, and the discrepancy factor is the geometrically averaged ratio of the higher to the lower among the modelled and observed averages, as also defined as in Table 5 of main article. Note that a perfect model would have the bias factor equal to 1 and the discrepancy factors equal to 1 + *s*, with *s* the average fractional error of the measurements.

campaign	area	*n*	Obs. [ppt]	A	B	C	C_VR
PEM-Tropics-A	Pacific	3390	496	0.99	0.75	0.73	0.90
PEM-Tropics-B	Pacific	1705	495	1.06	0.78	0.76	0.93
TRACE-A	Atlantic	697	407	0.85	0.66	0.65	0.82
PEM-West-A	W. Pacific	412	616	0.81	0.57	0.56	0.70
INTEX-A	N.E. Atlantic	1167	528	0.69	0.54	0.53	0.67
INTEX-B DC8	N.E. Pacific	2277	434	0.54	0.43	0.42	0.53
Polarstern 1994	Atlantic	~300	595	1.28	0.98	0.85	1.19
Polarstern 1996	Atlantic	~200	577	1.41	1.07	1.04	1.35
Mean bias factor				0.93	0.69	0.67	0.85
Mean discrepancy factor				1.27	1.47	1.51	1.32

Supplementary Table 6 | Global budget of methanol in model simulations. The simulations are defined in Table 4. Surface emissions include the biogenic source (104 Tg/yr), anthropogenic emissions (7.4 Tg/yr) and oceanic emissions (39.4 Tg/yr). Atomic chlorine concentrations in the marine boundary layer follow Allan et al.[18]

	**run A**	**run B**	**run C**	**run D**
		Sources (Tg/yr)		
Surface emission	162	162	162	162
CH_3O_2+RO_2	33.5	15.4	15.3	15.5
CH_3O_2+OH – direct	0	26.9	66.1	9.5
CH_3O_2+OH – indirect	0	17.1	50.6	0
CH_3O_2+OH – total	0	44.0	117	9.5
		Sinks (Tg/yr)		
CH_3OH+OH	119	137	189	112
CH_3OH+Cl	1.2	1.4	2.0	1.1
Wet deposition	3.6	4.0	5.5	3.4
Dry deposition	72	79	98	70
Burden (Tg)	2.9	3.2	4.7	2.8
Lifetime (days)	5.5	5.7	5.8	5.5

Supplementary Table 7 | Cartesian coordinates of all structures.

^{1}RC:

Total Energy=-265.9432068; ZPE=0.055009

C	0.000000	1.246316	0.000000
O	1.205606	0.463581	0.000000
O	0.961683	-0.803728	0.000000
H	-0.571536	1.011534	0.894941
H	-0.571536	1.011534	-0.894941
H	0.323491	2.281622	0.000000
O	-1.937803	-0.971075	0.000000
H	-1.016302	-1.292817	0.000000

^{1}PC:

Total Energy=-265.9551956; ZPE=0.054506

C	1.628742	0.487142	0.086932
O	1.168712	-0.783646	-0.118995
O	-1.421719	0.743819	-0.103887
H	2.715382	0.537990	0.160440
H	1.123806	0.954194	0.939204
H	1.286278	1.070935	-0.783745
O	-1.533053	-0.536806	0.111697
H	-0.609440	-0.872911	0.051992

^{1}TRIOX:

Total Energy=-265.9887127; ZPE=0.059977

C	1.435356	0.410874	0.096059
H	2.311358	0.332997	-0.542723
H	1.702136	0.198168	1.130030
H	0.998009	1.404183	0.008972
O	0.535346	-0.568846	-0.391069
O	-0.611205	-0.530560	0.403826
O	-1.397438	0.531250	-0.056213
H	-1.837260	0.144660	-0.824994

^{3}RC:

Total Energy=-265.943339; ZPE=0.054996

C	0.000000	1.244999	0.000000
O	1.203657	0.459504	0.000000
O	0.956810	-0.807082	0.000000
H	-0.572026	1.010940	0.894768
H	-0.572026	1.010940	-0.894768
H	0.325819	2.279619	0.000000
O	-1.930315	-0.959168	0.000000
H	-1.022975	-1.317523	0.000000

^{3}PC:

Total Energy=-265.9550979; ZPE=0.054645

C	-1.620170	0.479537	0.136608
O	-1.166546	-0.766724	-0.195293
O	1.425281	0.726117	-0.168542
H	-2.707935	0.527466	0.201976
H	-1.278012	1.135241	-0.682086
H	-1.124093	0.865690	1.032394
O	1.519636	-0.525857	0.182046
H	0.604094	-0.873903	0.082368

TS1:

Total Energy=-265.9418598; ZPE=0.053889

C	0.924374	0.994525	0.133180
H	1.758695	1.545473	-0.288133
H	0.949972	1.002052	1.219148
H	-0.027867	1.374248	-0.231848
O	1.079066	-0.356697	-0.322694
O	0.196118	-1.137687	0.200068
O	-2.054722	0.332451	-0.089009
H	-1.990743	-0.593450	0.194833

TS1':

Total Energy=-265.9427057; ZPE=0.055416

C	1.420651	0.584684	0.111956
H	2.246615	0.746873	-0.572723
H	1.773892	0.310812	1.102321
H	0.767360	1.452989	0.158800
O	0.666092	-0.502327	-0.434139
O	-0.317388	-0.805765	0.351457
O	-1.741125	0.568431	-0.016488
H	-2.172406	-0.101492	-0.566778

TS2:

Total Energy=-265.932444; ZPE=0.051199

C	0.183626	1.065231	0.187272
O	1.065537	0.126543	-0.372209
O	0.959439	-1.030595	0.189672
H	0.346887	2.012248	-0.314950
H	-1.450867	-1.032316	0.170972
H	0.295194	1.103715	1.266620
O	-1.948833	-0.240689	-0.093749
H	-0.902119	0.682894	-0.035986

TS3:

Total Energy=-265.9259064; ZPE=0.049529

C	-0.198421	1.097875	0.061624
O	-1.201578	0.107102	-0.111390
O	-0.794587	-1.109521	0.061318
H	-0.272795	1.532124	1.054738
H	1.448569	-1.025498	0.063326
H	-0.283783	1.817656	-0.745941
O	1.920621	-0.183186	-0.038708
H	0.902882	0.573301	-0.031622

TS4

Total Energy=-265.916138; ZPE=0.052988

Single point energy by M062x: -265.9454884

C	-1.666552	0.326826	-0.289243
O	-1.025081	-0.653799	0.389718
O	1.139055	0.478795	0.533503
H	-2.649726	-0.096651	-0.564113
H	-1.171243	0.608697	-1.228033
H	-1.877324	1.211401	0.325407
O	1.646492	-0.148756	-0.491416
H	1.613873	-1.094322	-0.252237

[1](CH_2O_2•H_2O):

Total Energy=-266.0126104; ZPE=0.057179

C	0.793908	1.017579	0.170024
O	1.076539	-0.074374	-0.329711
O	0.481950	-1.182473	0.187276
H	0.104686	1.062694	1.004290
H	-2.560461	-0.092907	-0.581365
H	1.273045	1.874761	-0.284049
O	-1.852207	0.205915	-0.010355
H	-1.230970	-0.542569	0.063292

[1](CH_3OH•O_2):

Total Energy=-266.0126473; ZPE=0.056862

C	-1.374636	0.706948	0.000963
O	-1.695905	-0.666812	-0.001252
O	1.876536	0.492893	-0.001642
H	-2.314006	1.253343	0.001616
H	-0.808074	0.997003	-0.887341
H	-0.808455	0.994225	0.890413
O	1.452090	-0.614970	0.001786
H	-0.883415	-1.175150	-0.001606

[1](CH_2O•H_2O_2):

Total Energy=-266.0555288; ZPE=0.057403

C	-1.596340	0.464701	-0.247000
O	-1.401304	-0.552288	0.359788
O	1.416818	0.661420	0.190978
H	-2.573628	0.967967	-0.205666
H	-0.808519	0.926558	-0.858820
H	2.056094	0.530290	0.898705
O	1.294679	-0.647222	-0.344416
H	0.422552	-0.908307	-0.003023

[3](CH_2O_2•H_2O):

Total Energy=-265.9623157; ZPE=0.053151

C	1.156770	-0.647117	0.000000
O	1.196824	0.736344	0.000000
O	0.000000	1.275023	0.000000
H	0.946549	-1.109066	0.950760
H	-2.734099	-1.029857	0.000000
H	0.946549	-1.109066	-0.950760
O	-1.777896	-1.096357	0.000000
H	-1.451051	-0.189386	0.000000

[3](CH_3OH•O_2):

Total Energy=-266.0351852; ZPE=0.056910

C	-1.385715	0.706532	0.000126
O	-1.699777	-0.668934	-0.000165
O	1.885765	0.494287	-0.000214
H	-2.328009	1.247868	0.000203
H	-0.820900	0.998294	-0.888681
H	-0.820962	0.997932	0.889091
O	1.460146	-0.614097	0.000233
H	-0.884913	-1.173340	-0.000201

CH_3O:

Total Energy=-115.0382929; ZPE=0.036716

C	0.010119	-0.575226	0.000000
H	-1.053141	-0.865822	0.000000
H	0.455737	-0.998747	0.904955
H	0.455737	-0.998747	-0.904955
O	0.010119	0.789334	0.000000

TS4':

Total Energy=-265.9435526; ZPE=0.055889

C	-1.505923	0.451994	0.145333
O	-0.866315	-0.647534	-0.388060
O	0.907001	-0.501250	0.449731
H	-2.463279	0.505311	-0.389523
H	-0.963605	1.379933	-0.055561
H	-1.709574	0.342559	1.212139
O	1.490564	0.514584	-0.150133
H	1.921996	0.133836	-0.931365

TS5:

Total Energy=-265.9427251; ZPE=0.051468

C	-1.388669	0.501933	0.062529
O	-1.166172	-0.780899	-0.074950
O	1.241883	0.729265	-0.100166
H	-1.888667	0.811486	0.986620
H	-1.742034	1.020545	-0.838874
H	-0.291592	0.942638	0.134274
O	1.345384	-0.545016	0.162965
H	0.885538	-1.013068	-0.559987

TS6:

Total Energy=-265.9528101; ZPE=0.052555

C	1.541284	0.515218	0.055923
O	1.133078	-0.768045	-0.090234
O	-1.456446	0.710857	-0.074946
H	2.374554	0.779508	-0.597550
H	1.721175	0.784079	1.102878
H	0.654025	1.129022	-0.239111
O	-1.377602	-0.565014	0.077809
H	-0.389693	-0.806299	0.097211

TS7:

Total Energy=-265.9489644; ZPE=0.050367;

C	1.503377	0.517957	0.013908
O	1.082756	-0.768775	-0.024022
O	-1.433471	0.704613	-0.019111
H	2.246112	0.745994	-0.753484
H	1.818248	0.832969	1.014793
H	0.593692	1.137464	-0.202060
O	-1.322059	-0.564033	0.013328
H	-0.296127	-0.798605	0.095744

CH_3OO:

Total Energy=-190.2031942; ZPE=0.043764

C	0.985396	-0.480608	0.000000
H	0.857582	-1.085162	0.893859
H	1.948356	0.019820	0.000000
H	0.857582	-1.085162	-0.893859
O	0.000000	0.559697	0.000000
O	-1.196987	0.069572	0.000000

OH:

Total Energy=-75.7303606; ZPE=0.008632

O	0.000000	0.000000	0.107877
H	0.000000	0.000000	-0.863016

OOH:

Total Energy=-150.9019604; ZPE=0.014657

O	0.054975	-0.597829	0.000000
H	-0.879607	-0.865134	0.000000
O	0.054975	0.705971	0.000000

Supplementary Note 1: Locating transition states TS1 and TS4

TS1 and **TS4** are crucial transition states that determine the dominate reactant pathways. With careful exploration, we located two transition states for **TS1** at M062x-D3/6-311++G(3df,3pd) level of theory. One (**TS1**) has a low imaginary frequency of -122 cm^{-1} that shows the reorientation of OH fragment and the formation of a new O–O bond, and the other transition state (**TS1'**) has an imaginary frequency of -266 cm^{-1} that mostly shows a character of breaking the O–O bond. Similarly, **TS4'** was successfully located with M06-2X-D3, which shows a pure O–O bond stretching character with an imaginary frequency of -397 cm^{-1}. The transition state **TS4** with a concerted mode (-208 cm^{-1}) that corresponds to the O–O bond breaking and OH fragment reorientation could be located using M06-D3 functional instead of M06-2X-D3.

For the open-shell singlet diradical molecules, it is known that density functionals might give poor results for unrestricted energies because of the admixture of multiple spin states[19]. On the singlet potential energy surface, **TS1** and **TS4** are two '*early*' transition states that leads to break the hydrogen bond in the reactant complex and the product complex, respectively. In the course of forming new O–O bond between the two radicals, the reaction might overcome another saddle point (**TS1'** or **TS4'**) to give **TRIOX,** or the appearance of these two transition states on the DFT potential energy surface might be an artefact due to the admixture of the singlet and triplet states.

In order to justify the 'real' transition state of **TS1** and **TS4**, we carried out a rigid scan from **RC** to **PC** that passing through all the 4 possible transition states **TS1**, **TS1'**, **TS4**, and **TS4'**. We interpolated 6 points for each of the linear conversion of **RC→TS1**, **TS1→TS1'**, **TS1'→TRIOX**, **TRIOX→TS4'**, **TS4'→TS4**, and **TS4→PC**, which give rise to 37 structures on the potential energy surface. Therefore, a rigid scan was performed at M06-2X-D3, CASPT2, and CCSD(T)-F12 level of theory, respective. The calculated potential energy surfaces are depicted in Supplementary Figure 3. The CCSD(T)-F12 relative energies agree reasonably well with the M06-2X-D3 energies for the triplet surface and for the region close to **RC** and **PC** on the singlet potential energy surface, supporting the use of this functional for geometry optimization. Large-active-space CASSCF and CASPT2 calculations (Supplementary Figure 3) strongly suggest **TS1'** and **TS4'** to be purely an artefact based on inaccurate description of the developing low-spin open-shell electronic structure, whereas the **TS1** and **TS4** are more reliable, and thus responsible for the formation and decomposition of **TRIOX**.

Supplementary Note 2: Intersystem crossing (ISC) of ^{1}PC and ^{3}PC

In the region of **PC**, there are in fact *four* close-lying singlet states, and *four* close-lying triplet states. This arises from the fact that both CH_3O and HO_2 are radicals with $^2A'$ and $^2A''$ states lying close in energy. In CH_3O, these states are very close in energy (ΔE of order << 1 kcal mol^{-1} at their respective minima, ΔE of about 2 kcal mol^{-1} vertical) and arise from Jahn-Teller splitting of the ^2E state. In HO_2, the difference in energy arising from splitting of the $^2\Pi$ state of linear HO_2 is larger.

At the M06-2X-D3/6-311++G(3df,3pd) structure for ^{1}PC, CASSCF(18,13)/cc-pVTZ state-averaged calculations indeed find 8 close-lying states, with relative energies of 0.0, 7.1, 21.8, 28.1 kcal mol^{-1} for singlet states and 0.0, 6.9, 21.7, 29.3 kcal mol^{-1} for triplet states. Presumably in each case the first two states correspond to the two Jahn-Teller states within the CH_3O moiety, with HO_2 in its ground state, and the two others correspond to the two Jahn-Teller states of CH_3O, with HO_2 in its excited state. Since these two higher states are much higher in energy, it is reasonable to consider the spin-orbit coupling of these two lower states of each multiplicity. Here we assume that the lowest singlet and second-lowest singlet as well as the lowest triplet and second-lowest triplet can interconvert readily due to the Jahn-Teller effects.

Now, the first singlet and triplet are degenerate, so the ^{1}**PC** minimum is effectively also the ^{3}**PC** minimum. So they can interconvert without a barrier. One very rough estimate of the rate constant for interconversion is to simply calculate the frequency associated with the coupling matrix element, as this corresponds to the frequency of Rabi cycling oscillation between the two states. However, because these two states both correspond to the same Jahn-Teller state of the CH_3O fragment (and the same state of the HO_2 system), then there is almost no spin-orbit coupling (SOC) between them, it comes out as about 0.4 cm^{-1}. There will also be some contribution from spin-spin coupling, but that will be also small.

However, the spin-orbit coupling between the lowest singlet state and the second-lowest triplet state (or between the second-lowest singlet and the lowest triplet) is expected to be much larger, as there is now an orbital angular momentum difference. Indeed, we find a coupling matrix element of 58 cm^{-1} in both cases. In reality, there will be some kind of barrier needing to be crossed to go from the minimum of the lowest singlet state in ^{1}**PC** to a region where the second triplet state is degenerate with it.

- We can estimate the rate for the interconversion of ^{1}**PC** and ^{3}**PC** in several ways:

(a) The MECP was optimized between ^{1}**PC** and ^{3}**PC** at the M06-2X-D3 level. This lies about 0.5 kcal mol^{-1} above ^{1}**PC**. However, this is the MECP between the lowest singlet and the lowest triplet. This is not exactly what we want to couple the lowest singlet state and the second-lowest triplet state (or between the second-lowest singlet and the lowest triplet).

(b) The second-lowest triplet and the second-lowest singlet lies 6.9 kcal mol^{-1} and 7.1 kcal mol^{-1} above the lowest singlet, respectively. Assuming harmonic potential energy surfaces, and knowing that all these states in fact correlate to the lowest singlet or triplet, we can guesstimate that the structure where the two lowest singlets and two lowest triplets are roughly degenerate will lie at about a quarter of the splitting, so at about 1.7 kcal mol^{-1}.

(c) The energy of the conical intersection between $^2A'$ and $^2A''$ states in methoxy is about 0.5 kcal mol^{-1} above the minima with a spin-orbit coupling constant of 160 cm^{-1} [20]. But in this case, there is a hydrogen bond (donated by HO_2) that splits the states a bit more.

Using a conservative relative energy for the 'true' MECP of 3 kcal mol^{-1} above the **^{1}PC** minimum, using the slopes of the singlet and triplet PESs at the **^{1}PC**/**^{3}PC** MECP, and the SOC of 58 cm^{-1} between the lowest singlet/second-lowest triplet, and an internal energy above the minimum of **^{1}PC** of 4400 cm^{-1}, corresponding to 12.5 kcal mol^{-1} above **^{1}PC**, corresponding to the relative energy of this point at the CCSD(T)-F12 level (-10.5 kcal mol^{-1}) + the ~2 kcal mol^{-1} of thermal energy in reactants, we can estimate the rate constant for **^{1}PC** → **^{3}PC** conversion, using the non-adiabatic RRKM theory method developed by J. N. Harvey[21-23]. We get 3.4 x 10^{12} s^{-1}, which is very close to the value given by Rabi-cycling method.

- Now, this value is subject to many approximations and assumptions. Some of these we can test, some we cannot:

(a) We assume that the NA-RRKM works (and that e.g. centrifugal effects are unimportant, the NA-RRKM is not J-resolved).

(b) We assume that the 'true' MECP lies about 3 kcal mol^{-1} above ^{1}PC. Here we can test this by changing the energy: raising the MECP or lowering it by 1 kcal mol^{-1} (keeping other parameters the same) decreases/increases the rate by about a factor of 2.

(c) We assume that the slopes on the potential energy surfaces at the correct MECP are similar to those at the incorrect, lowest-singlet-lowest-triplet MECP. The calculated rate constant obtained by making quite drastic changes to these slopes (factor of four change in slopes) also changes by about a factor of 2-3.

(d) We assume that the frequencies for the correct MECP are about the same as we get for the incorrect MECP.

On the basis of the convergence of the simple Rabi-cycling model, and NA-RRKM calculations, and their relative insensitivity to input parameters, we can be fairly confident that the rate-constant **^{1}PC** → **^{3}PC** at the internal energy for the reaction conditions is about 3×10^{12} s^{-1} with uncertainty by a factor of 2~3.

Supplementary Note 3: Frequencies of the structures along the PC dissociation pathway

For the variational RRKM calculations, we used the raw frequencies obtained from diagonalization of the Hessian at the structures obtained from a relaxed scan by constraining the dissociative coordinate (the O–H hydrogen bond in product complex, see Fig. 2). This leads to a total of 24 eigenvalues and eigenvectors at each point. Along the scan, the structures do not correspond to stationary points, so the frequencies and eigenvectors are not necessarily meaningful. Careful inspection of the eigenvectors shows however that they follow the physically-expected behaviour: 3 eigenvectors clearly correspond to translation of the whole system, 3 others clearly correspond to overall rotation, and one corresponds to the reaction coordinate, O–H stretching. The remaining 17 frequencies correspond to the expected conserved vibrations and transitional modes. The magnitudes of the frequencies vary in the expected way along the reaction path. Alternative use of projected vibrational frequencies[24] in which the directions corresponding to the three translations, the three rotations and the reaction path were projected out of the Hessian prior to diagonalization led to unphysical behaviour at points close to the **PC**. This appears to be due to mixing of the OO–H stretch mode and the breaking $CH_3O\cdots HOO$ hydrogen bond (the reaction coordinate).

For this reason, we used the unprojected frequencies for calculating the ZPVE to avoid the artificial initial discontinuity of -2.5 kcal mol^{-1} relative to the equilibrium ZPVE of the **PC** when using the projected frequencies. Second, for this specific case, it is mainly the OO–H stretch mode in the HO_2 moiety that leads to changes between unprojected and projected frequencies, as this mode has an important component along the RC at the early stages but not anymore in the "TS" region itself, and that anyway, near the TS, the H-O_2 stretch mode with its 10 kcal mol^{-1} quantum cannot be active at our low total distributable energy of only 8 ~ 9 kcal mol^{-1}. For these reasons, we used the unprojected frequencies for both the G and ZPVE in the k_{diss} calculations. Note that in the region of the variational TS, the unprojected and projected frequencies are in fact very similar to one another. We calculated the two k_{diss} also using projected frequencies for ZPVE and $G^{\mathrm{str}}(E_{\mathrm{v}} - E^{\mathrm{str}})$, finding k_{diss} values only 12 +/-3 % higher and a direct methanol yield only about 10% lower compared to the values using unprojected frequencie.

Supplementary Note 4: Sinks of stabilized trioxide CH_3OOOH

The important sinks for stabilized CH_3OOOH in the troposphere are likely: (i) thermal decomposition, (ii) gas-phase reaction with OH, (iii) gas-phase reaction with water dimer, and (iv) uptake by wet aerosol and cloud droplets (not necessarily in that order):

(i) Reaction (i) is the thermal analogue of conversion of **TRIOX** to **^{1}PC** followed by **^{1}PC** dissociation into $CH_3O + HO_2$ or decomposition into $CH_3OH + O_2$, discussed in the Kinetics Results subsection. The thermal rate coefficient for the rate limiting step TRIOX → ^{1}PC was evaluated by TST theory in the harmonic oscillator approximation using the energy and vibration/rotation data computed in this work: $k_{(i)}$(thermal) = $1.1\times10^{14}\times(T/298)^{3.5}\times\exp(-12130/T)$ s^{-1}. The product distribution at ambient temperatures was estimated at 0.2 ($CH_3OH + O_2$) + 0.8 ($CH_3O + HO_2$).

(ii) The CH_3OOOH + OH reaction is expected to proceed through a very stable, doubly-H-bonded 6-ring pre-reaction complex and a submerged TS, the latter also in view of the ~38 kcal mol^{-1} exothermicity of the abstraction of the terminal H, and should result finally in $CH_3O + O_2 + H_2O$[25]. By analogy with CH_3CHO + OH, with similar energetics and similar though less stable pre-reactive complex, the rate coefficient at 298 K can be roughly estimated at $k_{(ii)}$(298 K) = 2×10^{-11} cm^3 s^{-1} molecule $^{-1}$.

(iii) The reaction of CH_3OOOH with water dimer, by analogy with HOOOH + $(H_2O)_2$, is expected to proceed likewise through a very stable pre-reactive complex followed by a double H-shift through a TS lying about 5 kcal mol^{-1} above the reactants, to result in formation of $CH_3OH + O_2 + 2\ H_2O$[26]. Adopting a pre-factor equal to that for the analogous double-H-shift in the vinyl alcohol + formic acid reaction[16], the rate coefficient can be roughly estimated at $3\times10^{-15}\times\exp(-2500/T)$ cm^3 s^{-1} molecule $^{-1}$.

(iv) Given its ability to form stable, multiple H-bonded complexes with H_2O and H_2O clusters[26,27], CH_3OOOH should be highly hydrophilic and be readily taken up by aqueous aerosols and cloud droplets with an uptake coefficient of order 0.1, followed by its liquid-phase decomposition into $CH_3OH + O_2$[26,27].

Supplementary Note 5: Rotational effects

Rotation energy contributions to the effective potential energies[28,29] in the sequence of chemically activated steps have been examined as detailed here. The near-absence of collisions during the entire reaction sequence on the singlet surface entails the approximate conservation of the angular momentum D that determines the rotation energy and hence effective potential energy of the successive structures, depending on their moment(s) of inertia[27,28]. In the quasi-diatom approximation for each intermediate structure, the energy of rotation E'_{rot} about the 2 principal axes with highest moments of inertia I_B and I_C can be estimated as $E'_{rot} = D^2/2I$ with $I \approx (I_B \times I_C)^{1/2}$. The (constant) D^2 was obtained from the E'_{rot} and estimated $(I_B \times I_C)^{1/2}$ values of the variational entrance $\mathbf{TS_{in}}$ for CH_3O_2 + OH → ^{1}RC. With the average quasi-diatom $\langle E'_{rot} \rangle$ for the *thermal* $\mathbf{TS_{in}}$ equal to k_BT = 0.59 kcal mol^{-1} at 298 K, one can in this way estimate the $\langle E'_{rot} \rangle$ for all structures **^{1}RC**, **TS1**, **TS2**, **TRIOX**, **TS4**, **^{1}PC**, **TS5**, **TS6**, **TS7**, and the variational $\mathbf{TS_{diss}}$ at 0.69, 0.72, 0.78, 1.00, 0.65, 0.62, 0.76, 0.67, 0.71 and 0.52 kcal mol^{-1}, respectively. The $\langle E'_{rot} \rangle(T)$ are proportional to temperature T. At given total energy, the changes of the $\langle E'_{rot} \rangle$ of the successive activated reactants and transition states, relative to that of the initial reactant complex **^{1}RC**, impose opposite changes of their vibration energy E_v, and hence affect the rate coefficients of the activated reactions of **^{1}RC**, **TRIOX** and **^{1}PC**. The reactant complex **^{1}RC** serves here as reference compound because its vibration energy content (inherited form the thermal reactants CH_3O_2 + OH) can be estimated reasonably well (see Supplementary Note 6). For the case at hand, the effects of conservation of angular momentum on the effective potential energies are clearly modest, such that the various k's are only slightly or moderately affected, generally by only a few percent. The impact is among the largest for the important **TRIOX → ^{1}PC** reaction, increasing its rate by a factor 1.14 (at 298 K); note that the sometimes used "empirical" rotational correction[28] $[(I_A^{\neq} \times I_B^{\neq} \times I_C^{\neq}) / [(I_A \times I_B \times I_C)]^{1/2}$ would result here in a much too large factor of 1.65.

Supplementary Note 6: The average thermal energy < $E_{th,v}$> and the energy distribution of formation $F(E_{th,v})$

The thermal energy of the reactants at 298 K is made up of ~1.1 k_BT vibration energy of CH_3O_2 (= $\Sigma_i\{h\nu_i/k_BT/[\exp(h\nu_i/k_BT)$ -1]} × k_BT, summed over all vibration modes), 2 × 3/2 k_BT translation energy of the two reactants, 1 k_BT rotation energy of OH and 3/2 k_BT rotation of CH_3O_2. The CH_3O_2 vibration energy will be conserved in the complex **^{1}RC** as internal energy; 3/2 k_BT of translation remains translation energy of the center of mass, i.e. of the complex **^{1}RC**; the other 3/2 k_BT reactant translation can be considered as relative translation that goes mainly to internal energy of the complex and partly to its overall rotation (and contribute angular momentum); the 1 k_BT rotation of OH, with small angular momentum, can be considered as relative rotation that goes mainly to internal energy of the complex; the 3/2 k_BT rotation of CH_3O_2 is expected to become overall rotation of the complex for the larger part, while a fraction can go to internal energy. Therefore, at 298 K one expects an average internal (= vibration) energy $<E_{th,v}>$ around 3.6 k_BT or 2.1 kcal mol^{-1}, *versus* 3.0 k_BT or 1.8 kcal mol^{-1} energy of translation and overall rotation, with an expected error margin of ±0.5 k_BT each. The distribution function $F(E_{th,v})$ of formation can therefore be approximated by a Hinshelwood-type distribution[28] $F(E_{th,v}) = (E_{th,v}/k_BT\ )^{Seff\ -1} \times \exp(-E_{th,v}/k_BT\ )/(s_{eff}\ -1)!$ for a molecule with number of effective oscillators $s_{eff} \approx 3.6$ at 298 K and average $<E_{th,v}>$ at 298 K of ~2.1 kcal mol^{-1}. Similarly at 285 and 256 K, s_{eff} is estimated at 3.53 and 3.43, respectively, and the corresponding $<E_{th,v}>$ at ~2.0 and ~1.75 kcal mol^{-1}.

Supplementary References

1. O'Sullivan, D. W. *et al.* Distribution of hydrogen peroxide and methylhydroperoxide over the Pacific and South Atlantic Oceans. *J. Geophys. Res.* **104,** 5635-5646 (1999).
2. O'Sullivan, D. W. *et al.* Long-term and seasonal variations in the levels of hydrogen peroxide, methylhydroperoxide, and selected compounds over the Pacific Ocean. *J. Geophys. Res.* **109,** D15S13 (2004).
3. Singh, H. B., Brune, W. H., Crawford, J. H., Jacob, D. J. & Russell, P. B. Overview of the summer 2004 Intercontinental Chemical Transport Experiment–North America (INTEX-A). *J. Geophys. Res.* **111,** D24S01 (2006).
4. Singh, H. B., Brune, W. H., Crawford, J. H., Flocke, F. & Jacob, D. J. Chemistry and transport of pollution over the Gulf of Mexico and the Pacific: spring 2006 INTEX-B campaign overview and first results. *Atmos. Chem. Phys.* **9,** 2301-2318 (2009).
5. Junkermann, W. & Stockwell, W. R. On the budget of photooxidants in the marine boundary layer of the tropical South Atlantic. *J. Geophys. Res.* **104,** 8039-8046 (1999).
6. Weller, R., Schrems, O., Boddenberg, A., Gäb, S. & Gautrois, M. Meridional distribution of hydroperoxides and formaldehyde in the marine boundary layer of the Atlantic (48°N-35°S) measured during the Albatross campaign. *J. Geophys. Res.* **105,** 14401-14412 (2000).
7. Talbot, R. W. *et al.* Influence of biomass combustion emissions on the distribution of acidic trace gases over the southern Pacific basin during austral springtime. *J. Geophys. Res.* **104,** 5623-5634 (1999).
8. Singh, H. *et al.* Evidence from the Pacific troposphere for large global sources of oxygenated organic compounds. *Nature* **410,** 1078-1081 (2001).
9. Singh, H. B., Kanakidou, M., Crutzen, P. J. & Jacob, D. J. High concentrations and photochemical fate of oxygenated hydrocarbons in the global troposphere. *Nature* **378,** 50-54 (1995).
10. Fehsenfeld, F. C. *et al.* International Consortium for Atmospheric Reseach on Transport and Transformation (ICARTT): North America to Europe – Overview of the 2004 summer field study, *J. Geophys. Res.* **111**, D23S01 (2006).
11. Yang, M. *et al.* Atmospheric deposition of methanol over the Atlantic Ocean. *Proc. Nat. Acad. Sci.* **110,** 20034-20039 (2013).
12. Wisthaler, A., Hansel, A., Dickerson, R. R. & Crutzen, P. J. Organic trace gas measurements by PTR-MS during INDOEX 1999. *J. Geophys. Res.* **107,** 8024 (2002).
13. Karl, T., Hansel, A., Märk, T., Lindinger, W. & Hoffmann, D. Trace gas monitoring at the Mauna Loa Baseline Observatory using Proton-Transfer Reaction Mass Spectrometry. *Int. J. Mass spectrom.* **223-224,** 527-538 (2003).
14. Read, K. A. *et al.* Multiannual observations of acetone, methanol, and acetaldehyde in remote tropical atlantic air: implications for atmospheric OVOC budgets and oxidative capacity. *Environ. Sci. Technol.* **46,** 11028-11039 (2012).
15. Talbot, R. W. *et al.* Large-scale distributions of tropospheric nitric, formic, and acetic acids over the western Pacific basin during wintertime. *J. Geophys. Res.* **102,** 28303-28313 (1997).
16. Hoell, J. M. *et al.* Pacific Exploratory Mission in the tropical Pacific: PEM-Tropics A, August-September 1996. *J. Geophys. Res.* **104,** 5567-5583 (1999).
17. Peeters, J., Nguyen, V. S. & Muller, J. F. Atmospheric vinyl alcohol to acetaldehyde tautomerization revisited. *J. Phys. Chem. Lett.* **6,** 4005-4011 (2015).

18. Allan, W., Struthers, H. & Lowe, D. C. Methane carbon isotope effects caused by atomic chlorine in the marine boundary layer: Global model results compared with Southern Hemisphere measurements. *J. Geophys. Res.* **112,** D04306 (2007).
19. Ess, D. H. & Cook, T. C. Unrestricted prescriptions for open-shell singlet diradicals: using economical ab initio and density functional theory to calculate singlet-triplet gaps and bond dissociation curves. *J. Phys. Chem. A* **116,** 4922-4929 (2012).
20. Applegate, B. E., Barckholtz, T. A. & Miller, T. A. Explorations of conical intersections and their ramifications for chemistry through the Jahn–Teller effect. *Chem. Soc. Rev.* **32,** 38-49 (2003).
21. Harvey, J. N., Aschi, M., Schwarz, H. & Koch, W. The singlet and triplet states of phenyl cation. A hybrid approach for locating minimum energy crossing points between non-interacting potential energy surfaces. *Theor. Chem. Acc.* **99,** 95-99 (1998).
22. Harvey, J. N. & Aschi, M. Spin-forbidden dehydrogenation of methoxy cation: a statistical view. *Phys. Chem. Chem. Phys.* **1,** 5555-5563 (1999).
23. Harvey, J. N. Spin-forbidden reactions: computational insight into mechanisms and kinetics. *WIREs Comput. Mol. Sci.* **4,** 1-14 (2014).
24. Miller, W. H., Handy, N. C. & Adams, J. E. Reaction path Hamiltonian for polyatomic molecules. *J. Chem. Phys.* **72,** 99-112 (1980).
25. Denis, P. A. & Ornellas, F. R. Theoretical characterization of hydrogen polyoxides: HOOH, HOOOH, HOOOOH, and HOOO. *J. Phys. Chem. A* **113,** 499-506 (2009).
26. Koller, J. & Plesničar, B. Mechanism of the participation of water in the decomposition of hydrogen trioxide (HOOOH). A theoretical study. *J. Am. Chem. Soc.* **118,** 2470-2472 (1996).
27. Plesničar, B., Cerkovnik, J., Tekavec, T. & Koller, J. ^{17}O NMR spectroscopic characterization and the mechanism of formation of alkyl hydrotrioxides (ROOOH) and hydrogen trioxide (HOOOH) in the low-temperature ozonation of isopropyl alcohol and isopropyl methyl ether: water-assisted decomposition. *Chem. Eur. J.* **6,** 809-819 (2000).
28. Holbrook, K. A., Pilling, M. J. & Robertson, S. H. Unimolecular Reactions. John Wiley & Sons (1996).
29. Forst, W. Theory of Unimolecular Reactions. Academic Press, New York (1973).
